# Supplementary material for: Implementation of a Full Digital Workflow by 3D Printing Intraoral Splints Used in Dental Education: An Exploratory Observational Study with Respect to Students’ Experiences
Source: Dent J (Basel). 2022 Dec 26;11(1):5. doi: 10.3390/dj11010005 (PMC9858622; doi:10.3390/dj11010005)
Supplement: Supplementary file 1 [file dentistry-11-00005-s001.zip › Supplement S1- Questionnaire german.pdf]

Fragebogen: S D A E 1 2 3

	aktiver T.	passiver T.
Schienen-Code		

Datum:

UNIVERSITÄTS KLINIKUM TÜBINGEN

Studiencode: pse-2017

1. Fragebogen: Intraoralscan (S)

F1.01 **Die Handhabung des Scanners war**

einfach ├──────────┤ schwierig

F1.02 **Den zeitlichen Umfang des Scans bewerte ich als**

(Die gesamte Zeit für den Scan: Start: 1. Kieferbogen – Ende: Abschluss des Biss-Scan)

zügig ├──────────┤ langwierig

Real gemessene Zeit: ☐ Minuten

F1.03 **Wie viele Anläufe haben Sie benötigt?**

☐

F1.04 **Beim Vergleich der konventionellen Abformung mit dem intraoralen Scan – Welches Verfahren bevorzugen Sie:**

☐ intraoraler Scan ☐ konventionelle Abformung ☐ beide gleich

F1.05 **Waren Sie nach der Vorlesung mit Demo und ggf. dem Übungsscan für das selbstständige Scannen ausreichend vorbereitet?**

trifft voll und ganz zu ├──────────┤ trifft nicht zu

F1.06 **Wie würden Sie die Demo-Vorlesung zum IO-Scan bewerten?**

hilfreich ├──────────┤ überflüssig

F1.07 **Wie würden Sie den Scan im Studentenkurs im Hinblick auf die zahnärztliche Ausbildung bewerten?**

hilfreich ├──────────┤ überflüssig

F1.08 ***Wie kamen Sie mit der Scanzeit / Bilderanzahl zurecht?***

Sehr gut ├──────────┤ ungenügend

F1.09 ***Gab es Schwierigkeiten beim Umgang mit dem Scanner?***

(mehrere Antworten möglich) ☐ nein

☐ Bedienung der Software ☐ Bedienung des Scanners

☐ Reihenfolge beim Scannen (OK/UK/Biss) ☐ Scan über 1500 Bildern

Freitext:

F1.10 **Wie oft haben Sie den Scan für einen Einzelkiefer pausiert?**

☐ 0-3x ☐ 3-5x ☐ 5-10x ☐ mehr als 10x

aktiver Tandempartner

Bitte 2. Seite beachten!

Fragebogen:

S	D	A	E	1	2	3

	aktiver T.	passiver T.
Schienen-Code		

Datum:

UNIVERSITÄTS KLINIKUM TÜBINGEN

Studiencode: pse-2017

1. Fragebogen: Intraoralscan (S)
Seite 2

passiver Tandempartner

F1.11 **Wie empfanden Sie den Scanvorgang?**

angenehm |———————| ***unangenehm***

F1.12 **Empfanden Sie einen Würgereiz während dem Scanvorgang?**

keinen |———————| ***ausgeprägt***

F1.13 **Hatten Sie das Gefühl bei dem Scan den Mund sehr weit öffnen zu müssen?**

trifft nicht zu |———————| ***trifft voll und ganz zu***

F1.14 **Den zeitlichen Umfang des Scans bewerte ich als**

zügig |———————| ***langwierig***

F1.15 **Tragen Sie bereits eine Schiene / haben Sie in der Vergangenheit eine Schiene getragen?**

□ ja □ nein

F1.16 **Wie fühlten Sie sich nach dem Scan?**

(mehrere Antworten sind möglich)

□ unverändert

□ Muskelverspannung
□ trockener Mund
□ Geschmacksveränderung
□ Schmerzen im Mund
□ verändert beim zubeißen
□ Fremdkörpergefühl

Freitext:

Fragebogen: | S | D | A | E | 1 | 2 | 3 |

Schienen-Code	aktiver T.	passiver T.

Datum: ☐☐☐☐☐☐

UNIVERSITÄTS KLINIKUM TÜBINGEN

Studiencode: pse-2017

2. Fragebogen: Datenverarbeitung (D)

F2.01 **Die Konstruktion der Schiene war:**
(Bitte nur ein Kreuz)

☐ selbsterklärend ☐ herausfordernd ☐ schwierig ☐ sehr schwierig ☐ kann ich nicht beurteilen

F2.02 **Gab es Bereiche im Scan, die nicht ausreichend erfasst wurden?**

☐ nein ☐ ja – Regio:____

F2.03 **Waren Sie nach der Vorbereitung (Demonstration / Anleitung) ausreichend für das Schienendesign vorbereitet?**

trifft voll und ganz zu |————————————————| ***trifft nicht zu***

F2.04 **War Unterstützung notwendig?**

☐ nein ☐ etwas ☐ viel ☐ nur mit Hilfe möglich ☐ kann ich nicht beurteilen

F2.05 **Mussten Sie die Schienenkonstruktion mehrmals neu beginnen?**

☐ nein ☐ ja – Anzahl der Anläufe:______________ mal

F2.06 **Den Zeitlichen Umfang des Schienendesigns bewerte ich als:**

zügig |————————————————| ***langwierig***

Real gemessene Zeit: ☐ Minuten

F2.07 **Das Schienendesign traue ich mir alleine zu:**

trifft voll und ganz zu |————————————————| ***trifft nicht zu***

F2.08 **Sind Probleme aufgetreten?**

☐ nein ☐ ja *(-> Freitext)*

Freitext – ggf Rückseite benutzen

F2.09 **Ausdehnung der Schiene**

1) Fehlende Zähne bitte mit einem X markieren
2) Mit einem Stift die Ausdehnung der Schiene umranden

Bsp.:

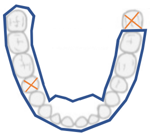

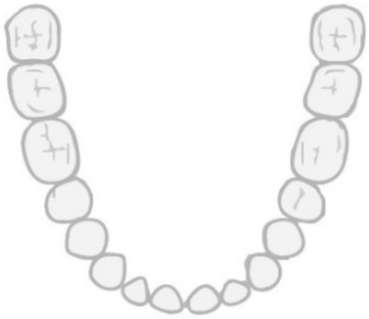

F2.10 **Welches Verfahren würden Sie für die Ausbildung bevorzugen?**

digital |————————————————| ***konventionell***

F2.11 **Haben Sie davor schon mit CAD-Designern gearbeitet?:** ☐ ja ☐ nein

aktiver Tandempartner

Fragebogen: | S | D | A | E | 1 | 2 | 3 |

Schienen-Code	aktiver T.	passiver T.

Datum: ☐☐☐☐☐☐

UNIVERSITÄTS KLINIKUM TÜBINGEN

Studiencode: pse-2017

3. Fragebogen: Ausarbeitung und Politur (A) Seite 2

F3.08 **Bitte kreuzen Sie die Instrumente an, die Sie zur Ausarbeitung angewendet haben (Mehrfachantwort möglich)**

Ausarbeitung & Entfernung Supportstruktur:

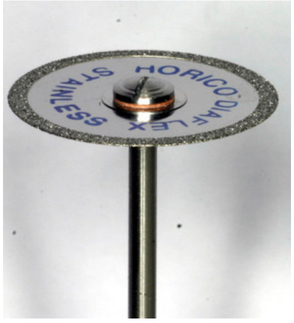

◇ Trennscheibe

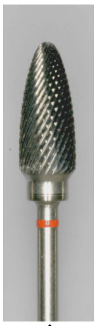

◇ kreuzverzahnte Fräse rot

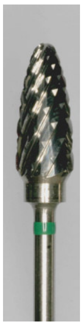

◇ kreuzverzahnte Fräse grün

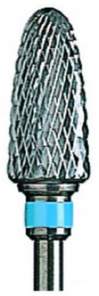

◇ kreuzverzahnte Fräse blau

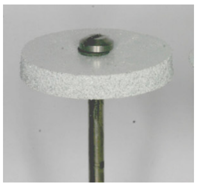

◇ Silikonrad

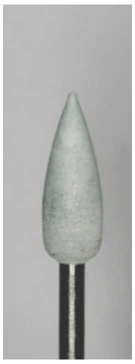

◇ Silikonpolierer

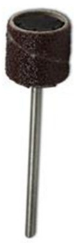

◇ Sandpapier

☐ andere:____

Politur:

☐ Ziegenhaarbürste ☐ Schwabbel ☐ Universalpoliturpaste
☐ Silikonpolierer ☐ Bimsmehl
☐ andere:____

☐ Handstück ☐ Poliermotor

F3.09 **Die Schiene ist bei der Ausarbeitung zu Bruch gegangen:**

☐ **Nein**
☐ Ja, bei:
☐ Support Entfernung ☐ Politur ☐ Ausarbeitung
☐ anderes:__________

F3.10 **Die Schiene werde ich mit folgender Fräse äquilibriert einschleifen:**

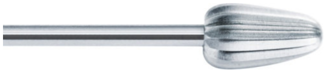

☐ MPS Fräse

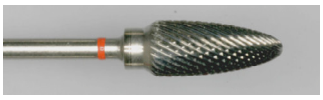

☐ Knospe

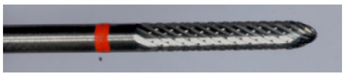

☐ gerade

☐ andere:____

aktiver Tandempartner

Fragebogen: | S | D | A | E | 1 | 2 | 3 |

Schienen-Code: aktiver T. ____ passiver T. ____

Datum: ____

Studiencode: pse-2017

3. Fragebogen: Ausarbeitung und Politur (A)

F3.01 **Eigenschaften des zu bearbeitenden Schienenmaterials:**

einfach |————————————————| **schwierig**

- ☐ Gewohnt
- ☐ ungewohnt
- ☐ Fräsen verstopfen
- ☐ Weich – viel Abtrag
- ☐ Hart – wenig Abtrag
- ☐ lässt sich gut polieren
- ☐ lässt sich schlecht polieren
- ☐ Schlierenbildung
- ☐ Kanten brechen ab
- ☐ geringer Arbeitsdruck nötig
- ☐ Hoher Arbeitsdruck nötig

F3.02 **Wie bewerten Sie den Arbeitsaufwand zur Ausarbeitung?**

einfach |————————————————| **schwierig**

F3.03 **Sind Probleme aufgetreten?**

☐ nein ☐ ja:

Freitext – ggf. Rückseite:

F3.04 **Wie beurteilen sie Ihr Endergebnis?**

sehr gut |————————————————| **schlecht**

Freitext:

F3.05 **Wie beurteilen Sie die Politur?**

sehr gut |————————————————| **schlecht**

F3.06 **Wie bewerten Sie den Aufwand für die Politur?**

sehr gering |————————————————| **sehr hoch**

F3.07 **Gibt es problematische Regionen an der Schiene, die Ihnen aufgefallen sind?**
(Bitte einzeichnen und kurz beschreiben)

Freitext – ggf. Rückseite:

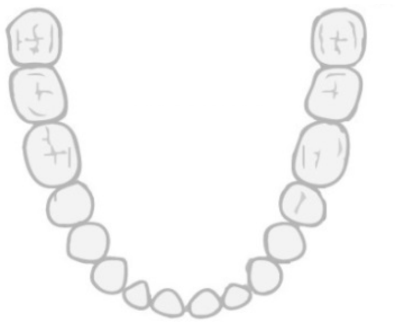

aktiver Tandempartner

Fragebogen: | S | D | A | E | 1 | 2 | 3 |

	aktiver T.	passiver T.
Schienen-Code		

Datum:

UNIVERSITÄTS KLINIKUM TÜBINGEN

Studiencode: pse-2017

4. Fragebogen: Anprobe und Einsetzen (E)

aktiver Tandempartner

F4.01 **Die initiale Passung der Schiene:**

sehr gut |——————| ***sehr schlecht***

□ kippelt □ Druck auf der Gingiva

□ spannt □ klemmt

Ergebnis: □ klinisch akzeptabel □ unzureichend

korrigierbar: □ ja □ nein

F4.02 **Der Halt der Schiene ist (gegen Ablösen aus Endposition):**

□ klinisch akzeptabel □ zu locker □ zu fest

korrigierbar: □ ja □ nein

F4.03 **Anzahl der statischen Okklusionskontakte initial (vor dem Einschleifen):**

Vorgehen:

Lassen Sie den Schienenträger 3mal auf Okklusionsfolie klappern.(li+re gleichzeitig, danach Front).

a) Zählen Sie anschließend alle Punkte auf der Schiene: ☐

*b) Bitte die entsprechenden Zähne mit Kontakt markieren **(x)***

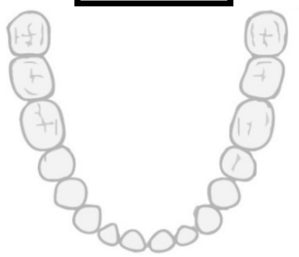

F4.04 **Wie bewerten Sie den Arbeitsumfang, die Schiene äquilibriert einzuschleifen?**

sehr gering |——————| ***sehr hoch***

□ einschleifen nicht möglich

Grund:________________

(ggf. Rückseite)

Zeitaufwand: ☐ Minuten

F4.05 **Wie beurteilen Sie das Endergebnis?**

sehr gut |——————| ***sehr schlecht***

□ unzureichend – nicht einsetzbar

F4.06 **Wie bewerten Sie den Gesamteindruck des Arbeitsablaufs (Scan- Design- einsetzen)?**

□ sehr gut □ gut □ zufriedenstellend □ ausreichend □ unbefriedigend

Bitte 2. Seite beachten!

Fragebogen: S D A E 1 2 3

aktiver T. passiver T.

Schienen-Code

Datum:

UNIVERSITÄTS KLINIKUM TÜBINGEN

Studiencode: pse-2017

4. Fragebogen: Anprobe und Einsetzen (E)
Seite 2

F4.07 **Merken Sie beim Einsetzen/Tragen der Schiene ein Spannungsgefühl?**

□ nein □ ja:

initial |————————| ***dauerhaft***

Regio:________________

F4.08 **Wie beschreiben Sie den Geschmack beim ersten Einsetzen der Schiene:**

□ neutral □ giftig □ chemisch □ süß □ sauer □ faulig □ sonstiges:__________

F4.09 **Wie ist das Gefühl mit der Schiene im Mund (ohne Okklusion)**

angenehm |————————| ***unangenehm***

korrigierbar: □ ja □ nein

Grund:

____

____

F4.10 **Ich habe Rauigkeiten auf der Schiene bemerkt:**

trifft nicht zu |————————| ***trifft voll und ganz zu***

korrigierbar: □ ja □ nein

F4.11 **Die Schiene zu tragen ist insgesamt (Tragegefühl + Okklusion):**

angenehm |————————| ***unangenehm***

korrigierbar: □ ja □ nein

Grund:

____

____

F4.12 **Ich möchte die Schiene zur Prophylaxe auch weiterhin tragen:**

□ ja □ nein

passiver Tandempartner
