# Supplementary material for: Implementation of a Full Digital Workflow by 3D Printing Intraoral Splints Used in Dental Education: An Exploratory Observational Study with Respect to Students’ Experiences
Source: Dent J (Basel). 2022 Dec 26;11(1):5. doi: 10.3390/dj11010005 (PMC9858622; doi:10.3390/dj11010005)
Supplement: Supplementary file 1 [file dentistry-11-00005-s001.zip › Supplement S2- Questionnaire english.pdf]

Questionnaire english

Questionaire	Type of question	Number	Question	VAS 0	VAS 100	variable 1	variable 2	variable 3	variable 4	variable 5	variable 6	variable 7	variable 8	variable 9	variable 10	variable 11
	**VAS**	**1.01**	**How do you evaluate the handling of the intraoral scanner?**	*simple*	*difficult*											
	**VAS**	**1.02**	**How do you rate the amount of time required for the scan?**	*swift*	*long-winded*											
	**Time**	1.02	real meassured time													
	**Number**	1.03	How many attempts did you need?													
	SC	1.04	When comparing the conventional impression with the intraoral scan - Which procedure do you prefer?			intraoral scan	conventional impression	both equal								
	**VAS**	**1.05**	**Were you adequately prepared for independent scanning after the lecture with a demo?**	*fully agree*	*totally disagree*											
	**VAS**	**1.06**	**How would you rate the demonstration and lecture on IO scanning?**	*helpful*	*unnecessary*											
	**VAS**	**1.07**	**How would you rate the integration of intraoral scanning in undergraduate dental education?**	*helpful*	*unnecessary*											
1. Intraoral scan	**VAS**	**1.08**	**How did you get along with the scan time/number of images?**	*very well*	*unsatisfactory*											
	MC, free text	1.09	Were there any difficulties in using the scanner?			none	Software operation	Scanner operation	Scan sequence (Upper jaw/lower jaw/ bite)	scan over 1500 pictures						
	SC	1.10	How often did you pause the scan for a single jaw scan?			0-3x	3-5x	5-10x	more than 10x							
	**VAS**	**1.11**	**How did you feel during the scanning process?**	*pleasant*	*unpleasant*											
	**VAS**	**1.12**	**Did you feel a gagging sensation during the scanning process?**	*none*	*pronounced*											
	**VAS**	**1.13**	**Did you have the feeling that you had to open your mouth very wide during the scan?**	*fully agree*	*totally disagree*											
	**VAS**	**1.14**	**How do you rate the amount of time required for the scan?**	*swift*	*long-winded*											
	MC, free text	1.16	How did you feel after the scan?			Without change	muscle strain	mouth pain	dry mouth	changed okklusion	change in tase	foreign body sensation				
	SC	2.01	The construction of the splint was:			self-explaining	challenging	difficult	exceedingly difficult							
	SC, text	2.02	Were there areas in the scan that were not adequately captured?			no	yes - region:									
	**VAS**	**2.03**	**Were you adequately prepared for the splint design after preparation (demonstration / instruction)?**	*fully agree*	*totally disagree*											
	SC	2.04	Was assistance necessary?			no	slightly	a lot	only possible with assistance							
2. CAD	SC, Number	2.05	Did you have to restart the splint design several times?			no	yes - number of attempts:									
	**VAS**	**2.06**	**How do you rate the time required for the splint design (CAD)?**	*swift*	*long-winded*											
	Time	2.06	real meassured time													
	**VAS**	**2.07**	**I can perform the splint design independently**	*fully agree*	*totally disagree*											
	SC, free text	2.08	Have any problems occurred			no	yes									
	**VAS**	**2.10**	**Which method would you prefer for the training?**	*digital*	*conventional*											
	SC	2.11	Have you worked with CAD designers before?			yes	no									
	**VAS**	**3.01**	**Properties of the splint material to be processed**	*simple*	*difficult*											
	SC	3.01	Properties of the splint material to be processed			familiar	unfamiliar	milling clog	soft - much remove	hard - less remove	polishes well	does not polish well	streaking	edges breaking off	low working pressure required	high working pressure required
	**VAS**	**3.02**	**How do you evaluate the time and effort for the elaboration?**	*simple*	*difficult*											
3. Finishing	SC, free text	3.03	Have problems occurred			no	yes									
	**VAS, free text**	**3.04**	**How do you rate your final result?**	*very good*	*very poor*											
	**VAS**	**3.05**	**How do you rate the polishing?**	*very good*	*very poor*											
	**VAS**	**3.06**	**How do you rate the effort for polishing?**	*very low*	*very high*											
	**Free text**	3.07	Are there any critical areas of the splint that you have noticed?			*no*	*yes - free text*									
	**VAS**	**4.01**	**The initial fit of the splint is:**	*very good*	*very poor*											
	**MC**	4.01	The initial fit of the splint is:			*wobbling*	*tension*	pressure on the gingiva	jamming							
	**SC | SC**	4.01	The initial fit of the splint is:			clinical acceptable	clinical unacceptable	correctable yes / no								
	**SC | SC**	4.02	The retention of the splint (against detachment from end position)			clinical acceptable	too loose	too tight	correctable yes / no							
	**Number**	4.03	Number of initial static contacts (before grinding)													
	**VAS**	**4.04**	**How do you rate the effort to obtain an equilibrated bite plane?**	*very little*	*very high*			not possible								
	**Time**	4.04	How do you rate the effort to obtain an equillibrated bite plane? (Duration in Minutes)													
4. Insertion	**VAS**	**4.05**	**How do you rate the final result?**	*very good*	*very poor*	inadequate - not insertable										
	**SC**	4.06	How do you rate the overall workflow with scanning, designing, finishing, and inserting the splin			very good	good	satisfactory	adequate	inadequate						
	**VAS, Text**	**4.07**	**Do you notice a feeling of tension when inserting/wearing the splint**	*initial*	*permanent*											
	**MC**	4.08	How do you describe the taste when you first insert the splint:			neutral	toxic	chemical	sweet	sour	foul	other				
	**VAS, SC, Text**	**4.09**	**How is the feeling with the splint fitted in the mouth (without occlusion)**	*comfortable*	*uncomfortable*	correctable yes / no										
	**VAS, SC, Text**	**4.10**	**I have noticed roughness on the splint**	*fully agree*	*totally disagree*	correctable yes / no										
	**VAS, SC, Text**	**4.11**	**Wearing the splint is (wearing comfort + occlusion):**	*comfortable*	*uncomfortable*	correctable yes / no										
