# Supplementary material for: Implementation of a Full Digital Workflow by 3D Printing Intraoral Splints Used in Dental Education: An Exploratory Observational Study with Respect to Students’ Experiences
Source: Dent J (Basel). 2022 Dec 26;11(1):5. doi: 10.3390/dj11010005 (PMC9858622; doi:10.3390/dj11010005)
Supplement: Supplementary file 1 [file dentistry-11-00005-s001.zip › Supplement S3- Manual ExoCad VALETTA splint design 2.2.pdf]

<u>Allgemeine Voreinstellungen im CAD:</u>

Werkzeuge -> Einstellungen

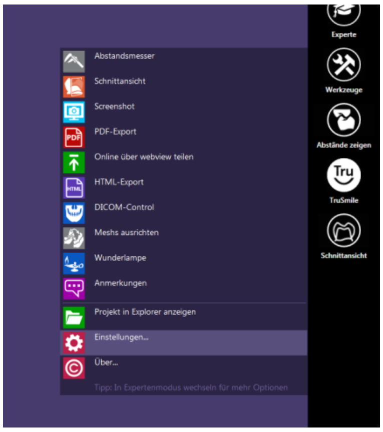

Hacken wie abgebildet setzen:

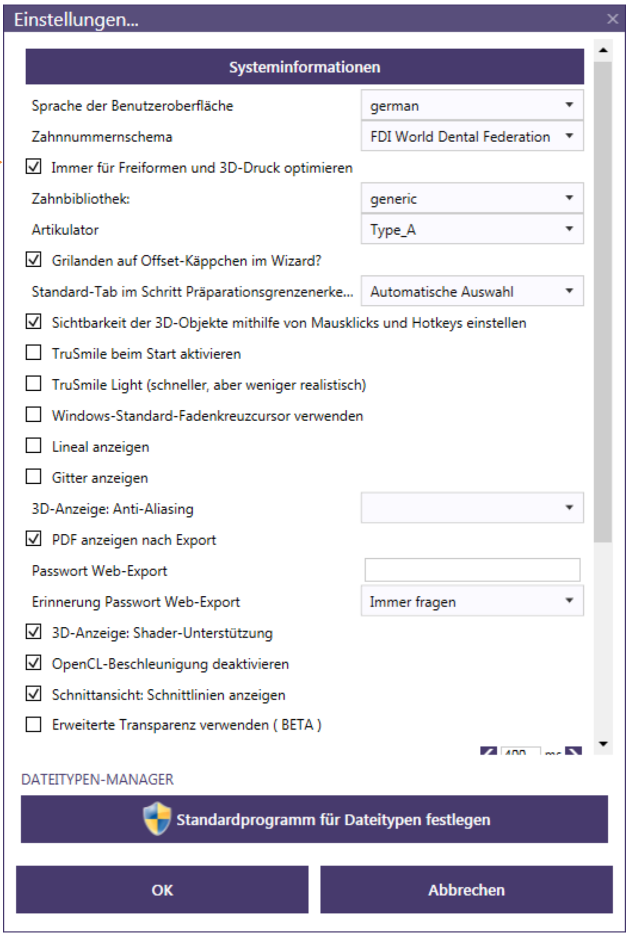

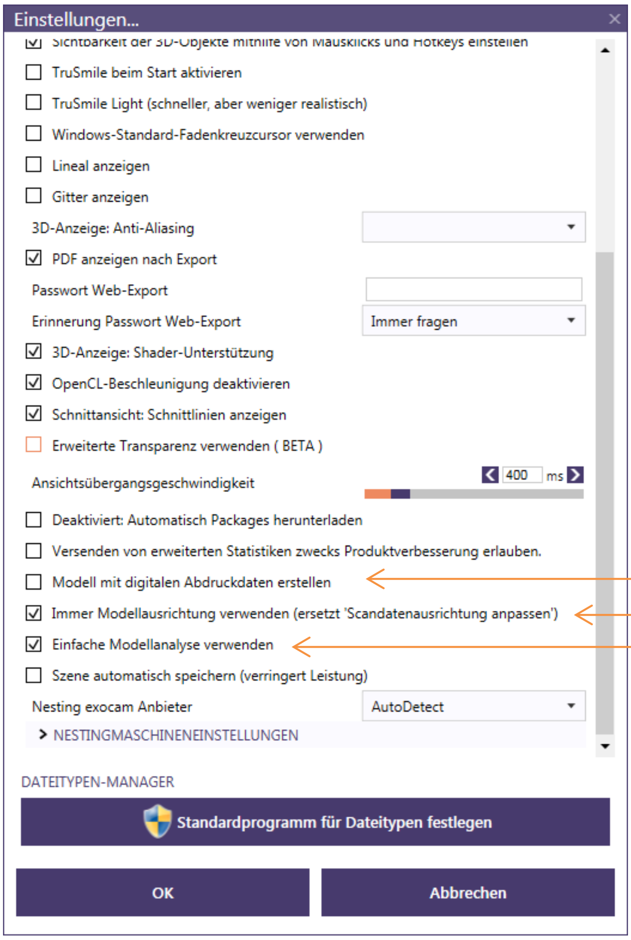

Allgemeine Bedienung im Designer:

<u>Ansicht</u>

rechte Maustaste gedrückt halten: **drehen der Ansicht / Ausrichten des Modells**

rechte + linke Maustaste gleichzeitig gedrückt halten: **bewegen des Modells**

Mausrad drehen: **zoomen**

Mausrad klicken: **neuen Fixpunkt setzen, um den sich das Modell dreht (bei gedrückter rechter Maustaste)**

<u>Modellieren</u>

Linke Maustaste: **Auftragen +**

Linke Maustaste + 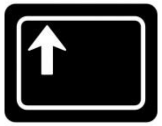 **gedrückt halten:** **Abtragen -**

Fenster im ExoCAD CAD-Designer

Speichern *für späteres weiterarbeiten*

Zeigt an, was momentan eingeblendet wird

- ➔ **Durch den Schieberegler kann die Intensität bestimmt werden**
- ➔ **Durch klicken auf den Kasten ☐ : Objekt ein-/ausblenden**

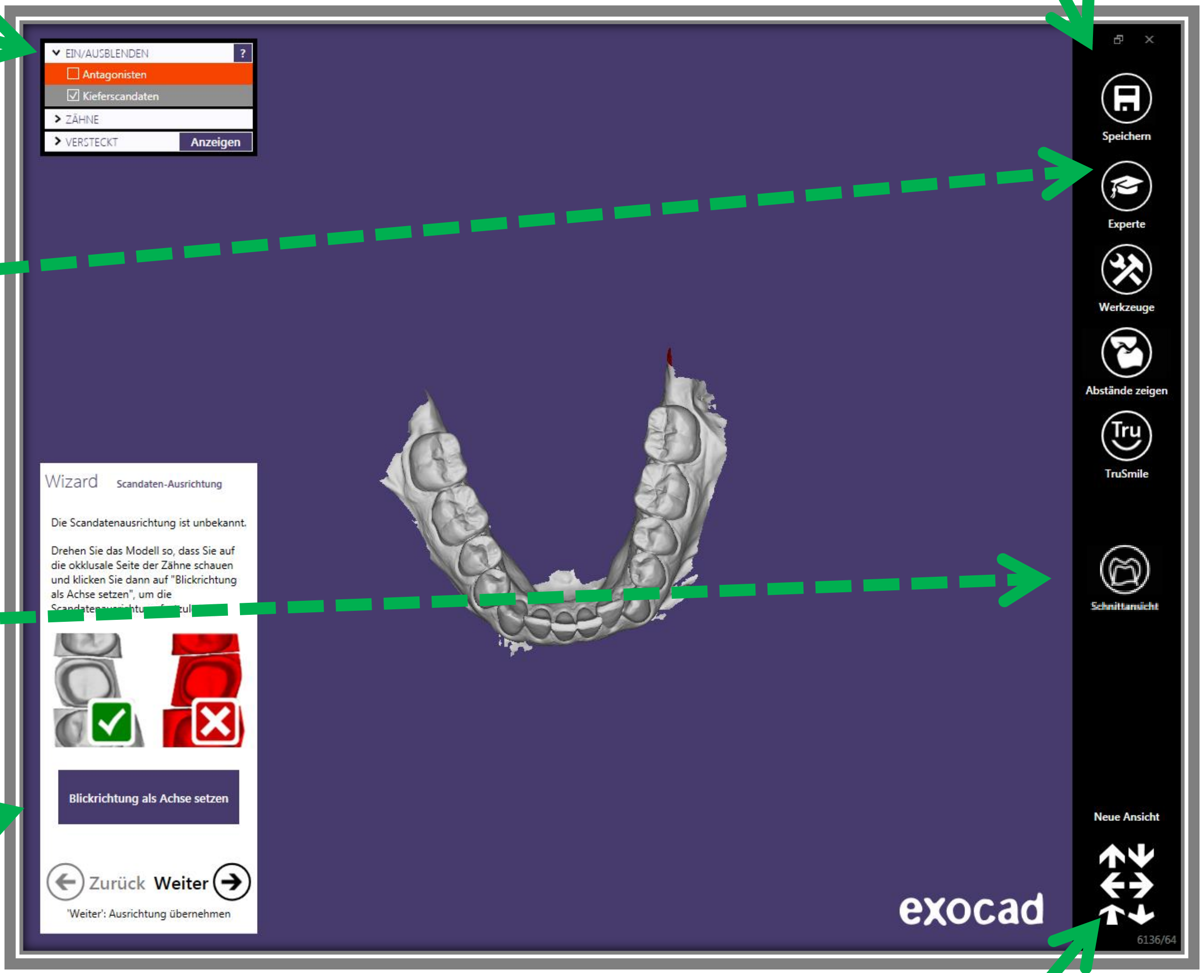

Experte/Wizard:

Ermöglicht zusätzliche Einstellungen im <u>Expertenmodus</u> bzw. startet den <u>Wizard</u> erneut

TruSmile – auf realistische Ansicht umschalten

Schnittansicht durch die Modelle

(Öffnen und Schließen durch Klick auf die Schaltfläche – ermöglicht das Messen von Abständen)

Wizard – leitet durch das Schienen-Design

Definierte Ansichten. Durchschalten durch Klick auf die Pfeile

Anleitung Schienenherstellung EXOCAD V2.2

Diese Anleitung führt Sie Schritt für Schritt durch die Herstellung einer UK-Schiene mit adjustierter, äquilibrierter Aufbissfläche. Begleitend dazu steht ein Video mit dem Behandlungsablauf zur Verfügung.

Zeit messen - ab jetzt ☺

1) Starten von ExoCad vom Desktop aus: 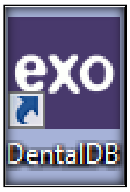

1) Auftrag in ExoCad anlegen:

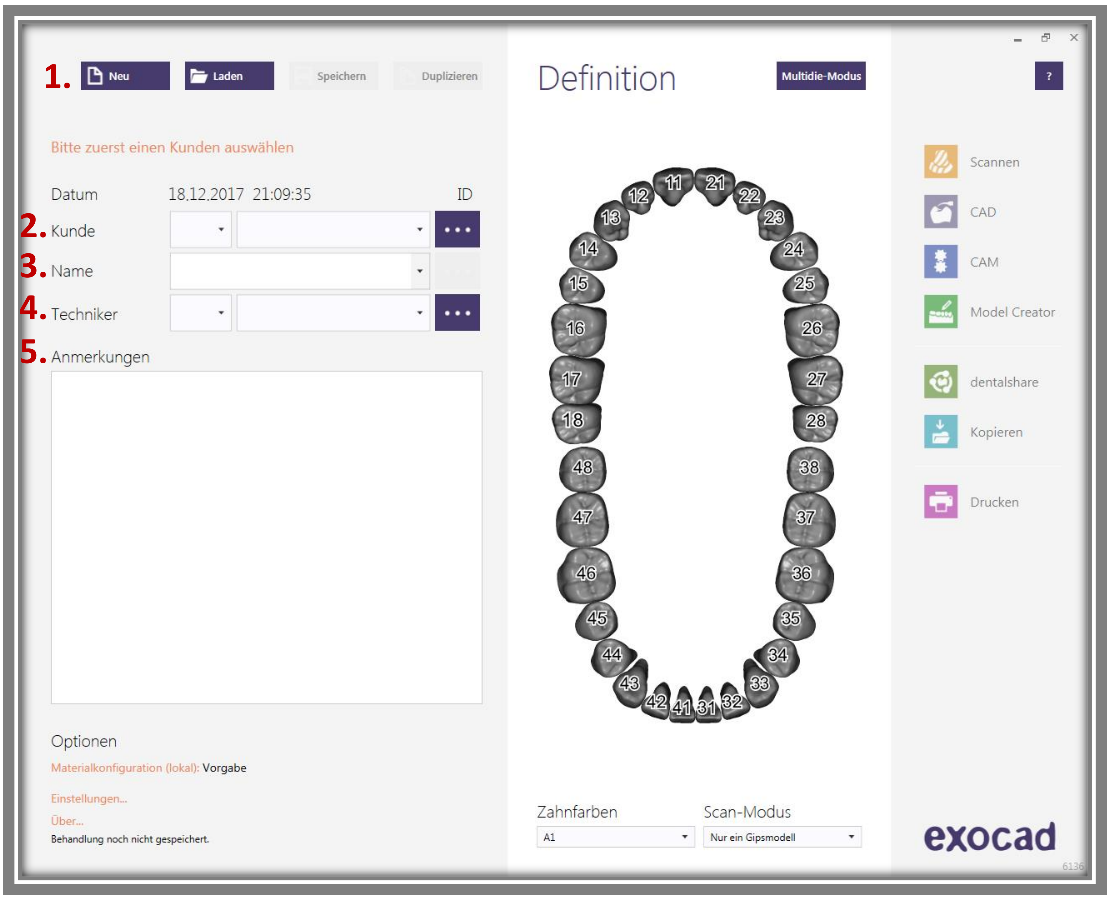

1) Auf **Neu** klicken
2) Kunde: 00001 - Studentenkurs
3) Name: Name des Studierenden, der die Schiene bekommt
4) Techniker: 00001 / Default
5) Anmerkungen: Name des Studierenden, der die Schiene designt

Art der Versorgung auswählen:

6) Zahn 48 im Unterkiefer anklicken- >

➔ **Aufbissschiene** auswählen

7) Mindestdicke: **0,5mm** **-> Bestätigen mit OK**

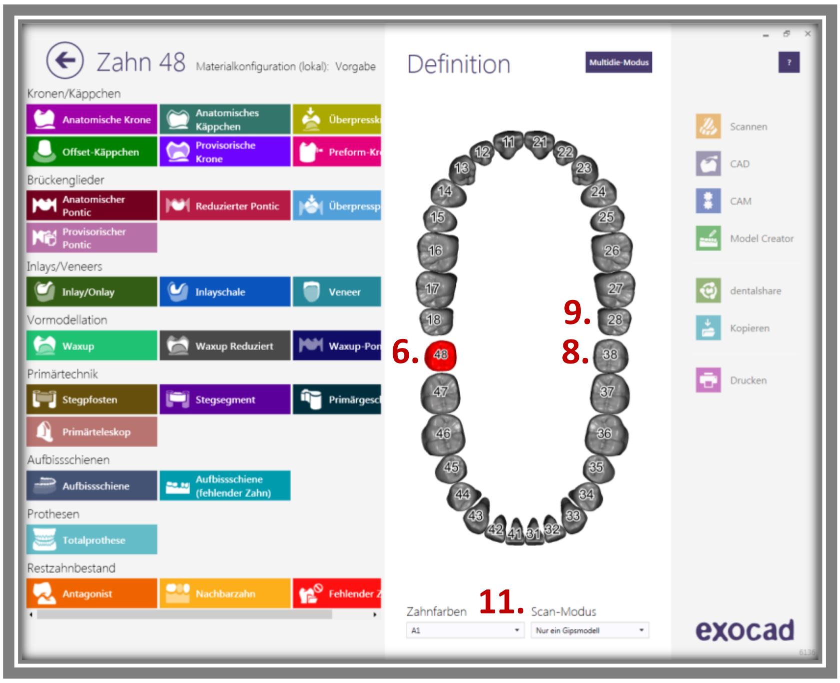

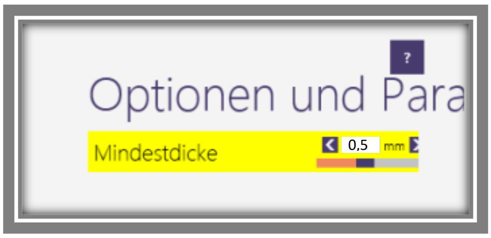

8) Mit gedrückter *Shift -Taste* Zahn 38 anklicken

9) Zahn 18 anklicken

➔ **10) Antagonist** auswählen -> Bestätigen mit **OK**

➔ Mit gedrückter *Shift-Taste* Zahn 28 anklicken

11) Scan-Modus: ***Zwei Gipsmodelle in Artikulator A***

12) Speichern!

13) Starten des Schienen.-Designers **(CAD)**

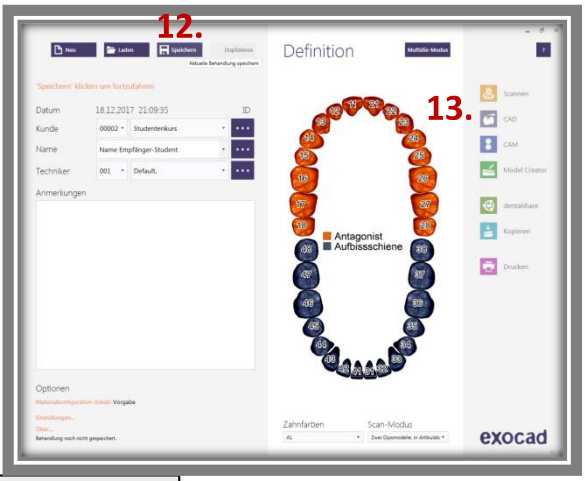

CAD lässt sich erst starten, sobald der Auftrag vollständig angelegt und abgespeichert wurde

CAD Designer:

Das Starten kann bis zu 4 Minuten dauern – Warnmeldungen ignorieren

Scans laden:

➔ **Achtung**! Es werden immer die zuletzt geladenen Scans angezeigt.
Zurück auf den ***Desktop -> Digitale-Lehre*** und die entsprechenden Daten wählen

Reihenfolge beachten!

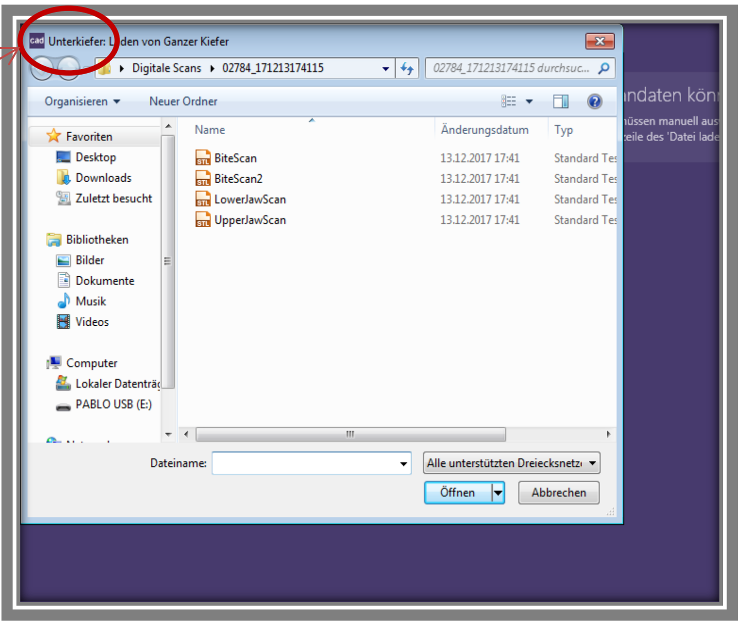

1. **LowerJawScan** auswählen -> Öffnen
2. **UpperJawScan** auswählen -> Öffnen

<u>Die Scans werden nun geladen und der Wizard führt automatisch durch die Schienen-Herstellung</u>

Falsche Scans geladen oder vertauscht? CAD schließen und aus dem Auftragsfenster CAD neu Starten (Punkt 13)

2) Modell erstellen:

1) Wählen Sie im Fenster **Modelltyp:**

Plateless model with extra dies

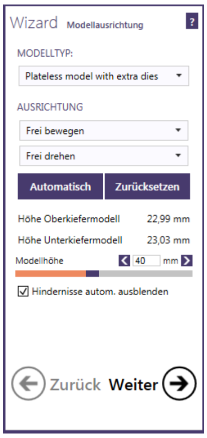

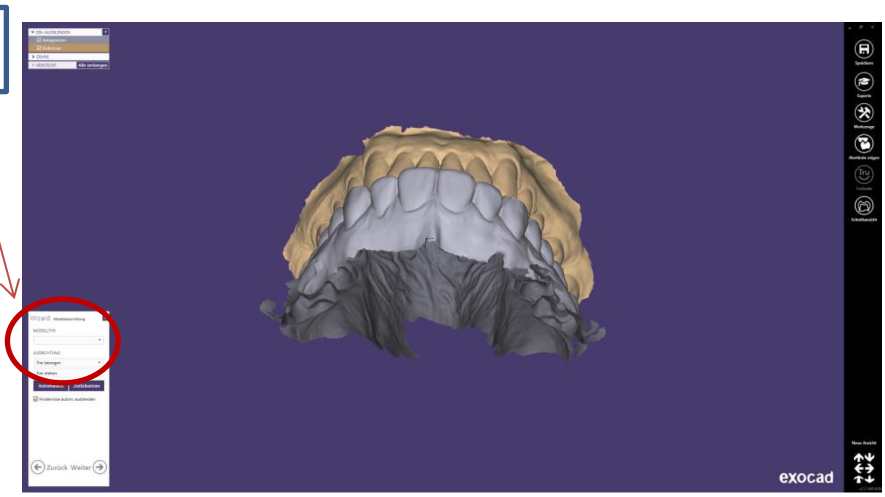

2) Richten Sie die Scans zwischen den Basen-Platten**(OK blau** / **UK grün)** aus, falls dies nicht automatisch geschehen ist.

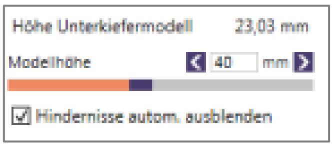

Passen Sie ggf. die Modellhöhe an.

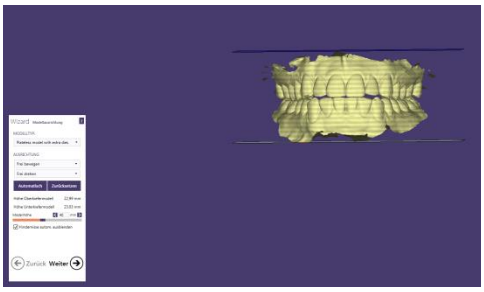

Manuelles Ausrichten der Modelle:

Modell bewegen mit Linker Maustaste gedrückt halten

Modell drehen durch **halten** der **STRG Taste**

(Drehen durch *klicken und halten* der Pfeile)

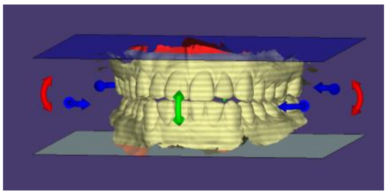

Feritg? Klicken Sie auf

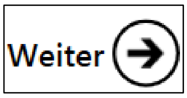

3) **Warnmeldung** mit **Ja** bestätigen

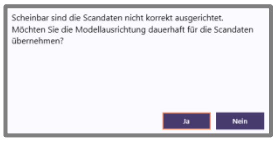

4) Modell zurecht schneiden:

☆ **Ziel:** alle Bereiche entfernen die in den Gegenkiefer ragen und die Artikulation stören (Trigonom + Tuber -distal der 7er sowie Umschlagfalte)

a. Antagonist ausblenden (auf ☐ klicken um entspr. Kiefer ein- oder auszublenden) zur einfacheren Orientierung

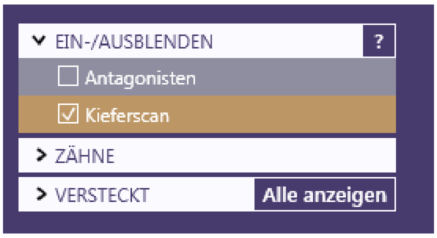

b. Mit linker Maustaste Punkte setzen und Bereiche markieren (Abbruch mit rechtsklick)

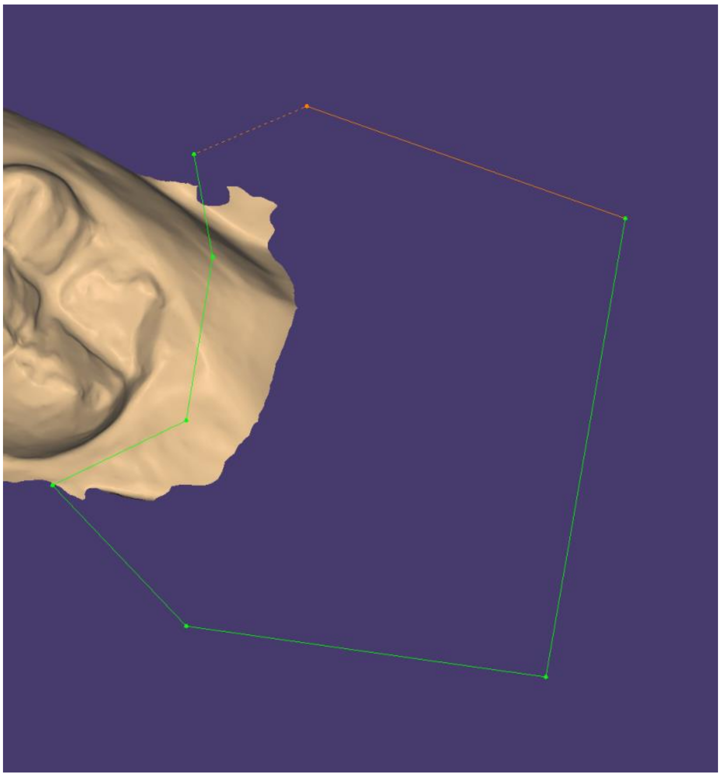

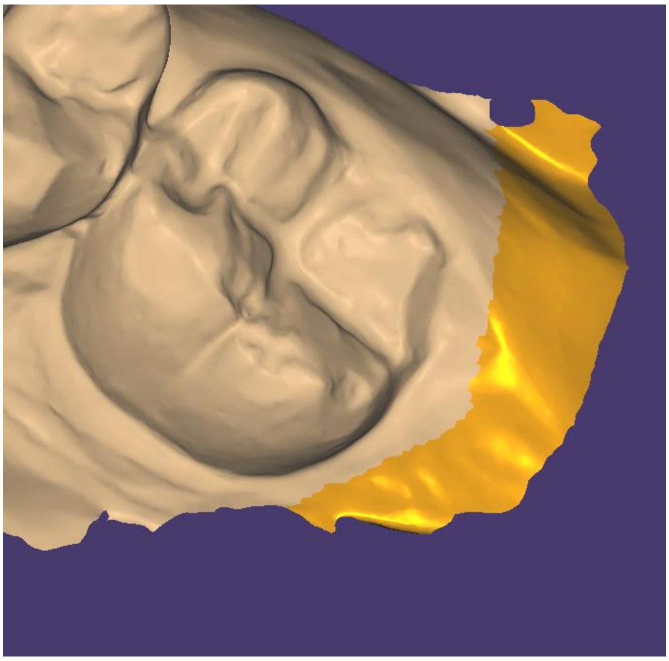

Letzer Punkt: Doppelklick um umrandete Fläche auszuwählen

c. Entfernen der Fläche durch Klick auf **Löschen**:

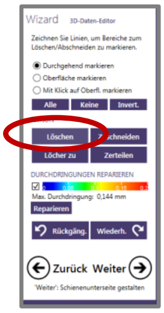

➔ ***Wiederholen***, bis alle Flächen entfernt sind.

d. Anschließend den **Oberkiefer** auf gleiche Art vorbereiten (UK ausblenden – OK einblenden)

e. Abschließende Kontrolle, dass alle Störstellen entfernt sind:

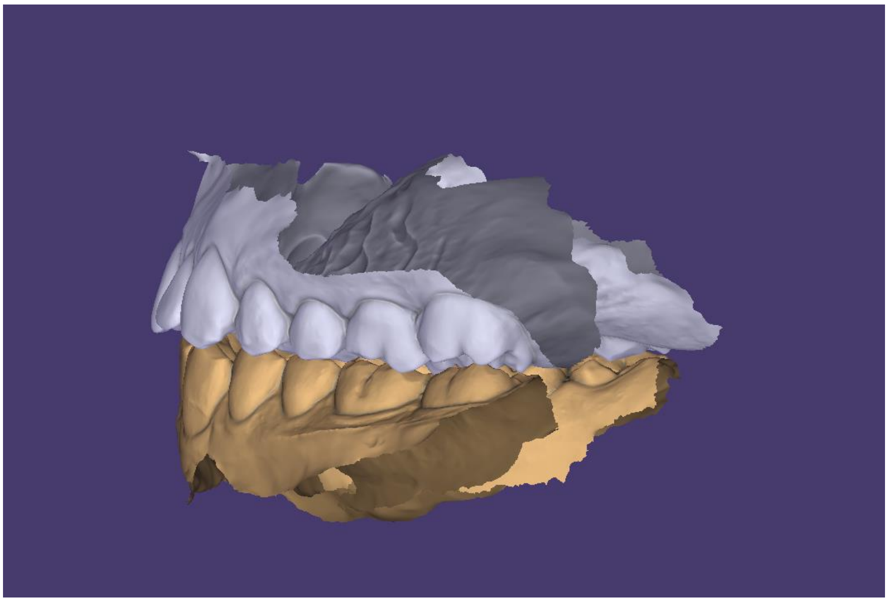

f. Klicken Sie auf Löcher zu (Grenzwert: 5)

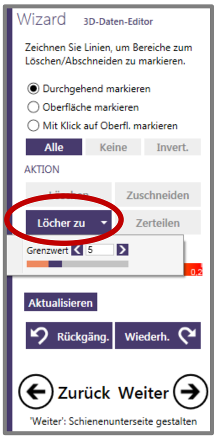

g. Abschluss - **Klicken Sie auf:** *Durchdringung nochmals prüfen*

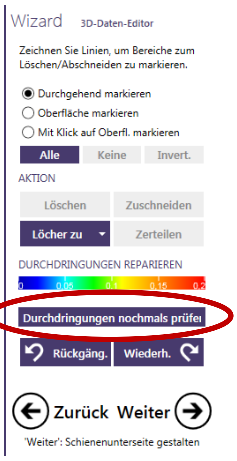

5) Keine Fehler? -> Vorbereitungen sind abgeschlossen. Klicken Sie auf

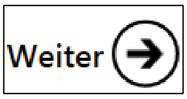

3) Schienendesign

1) Einschubrichtung setzen:
Halten Sie die rechte Maustaste gedrückt um das Modell auszurichten.
☆ *Ziel: Einschubrichtung mit möglichst wenig Unterschnitt – FZ sollten zervikal gerade noch sichtbar sein*

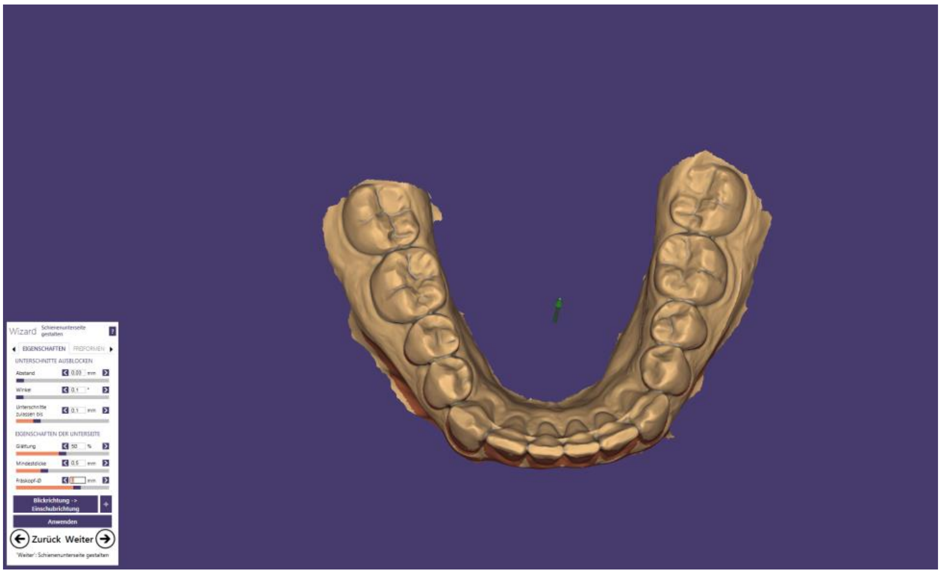

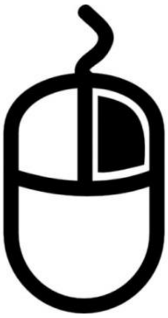

2) Einschubrichtung übernehmen: **Klick auf**:

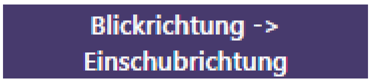

3) Parameter zum Ausblocken einstellen:

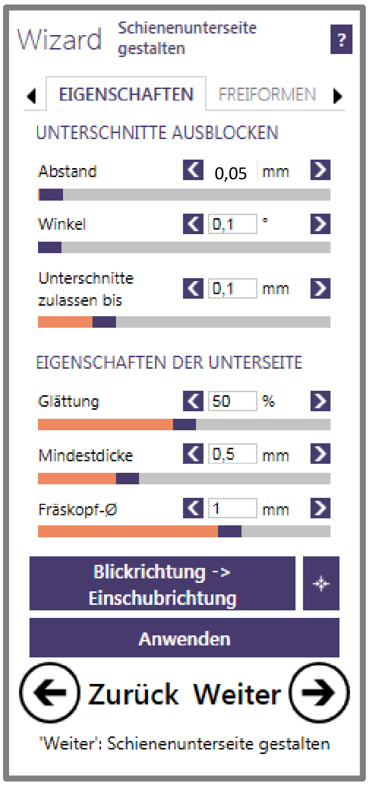

4) Auf **Anwenden** klicken

☆ *Zur weiteren Bearbeitung ist es einfacher, die Ausblockung auf 100% zu schalten*

5) Einblenden der Ausblockung: Regler ganz nach rechts auf 100%:

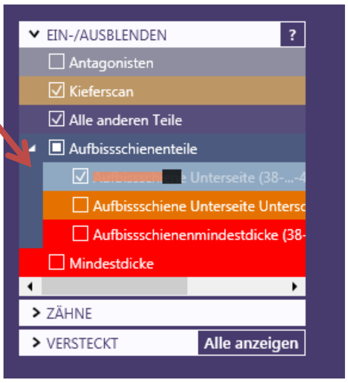

6) Klicken Sie auf den Reiter Freiformen

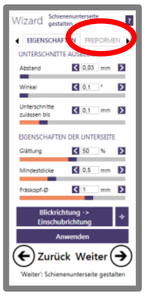

7) Wählen Sie Auf-/abtragen

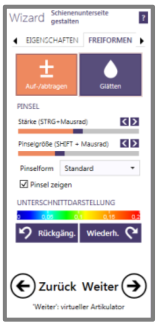

8) Blocken Sie den Rest des Modells ggf. von Hand aus:

a. Retainer

b. Verschachtelte Zähne

c. 3er Vestibulär-zervikal ganz leicht (sonst spätere höhere Spannung in der Schiene)

d. Alle Unterschnitte im Bereich des lingualen „Schilds“

e. Unterschnitte im FZ-Bereich

☆ *Bsp. Retainer:*

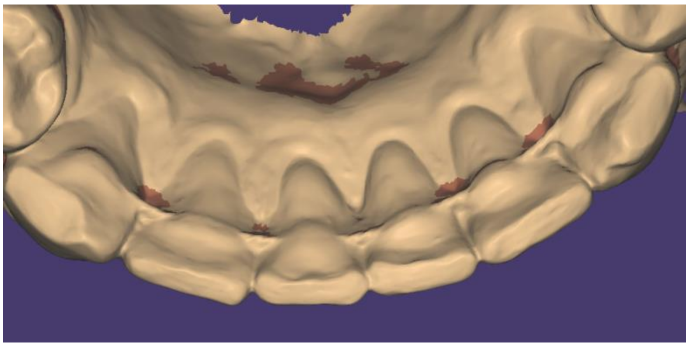

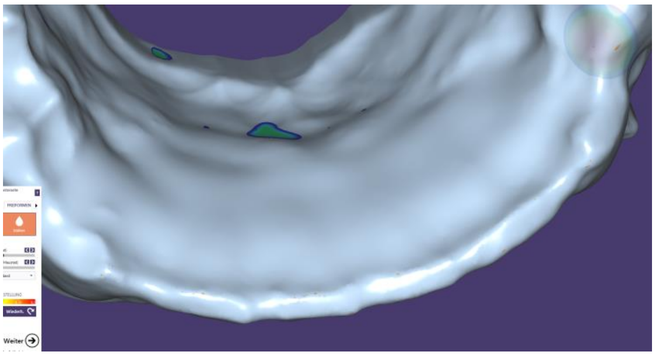

9) Modell leicht glätten (v.a. interdental und die zusätzlich ausgeblockten Bereiche)

10) Okklusal freilegen: Entfernen des „Wachs“ durch halten der *Shift-Taste* 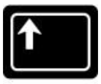 *auf den Molaren, Prämolaren und der Inzisalkanten*

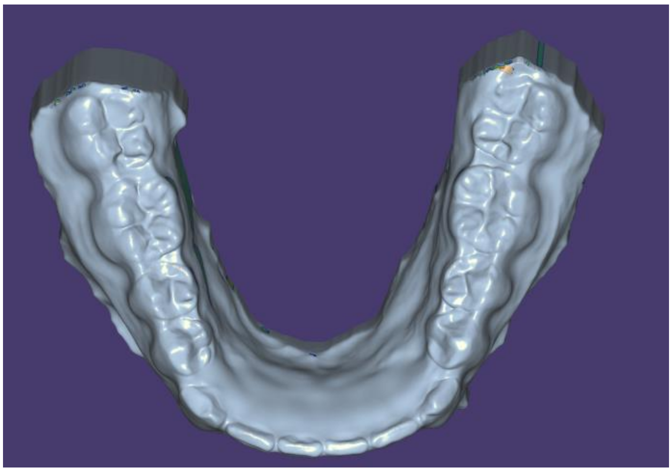

11) Fissuren okklusal vorsichtig mit glätten-Tool entfernen

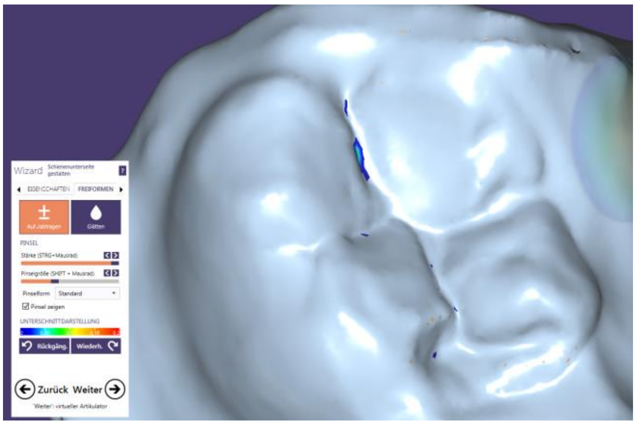

Fertig? Klicken Sie auf 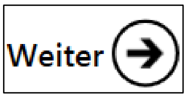

12) Virtueller Artikulator:

Klicken Sie auf **„Virtuellen Artikulator jetzt starten“**

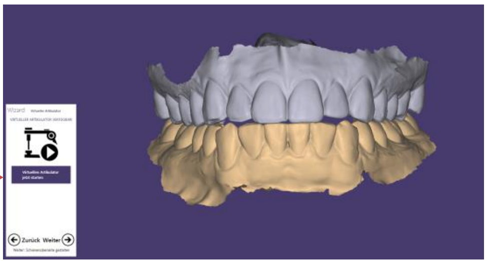

13) Warnmeldung bestätigen durch klicken auf **„Kieferabstand übernehmen“**

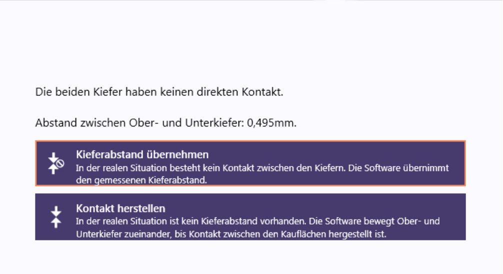

14) Klicken sie auf „**Virtuell Einartikulieren**“

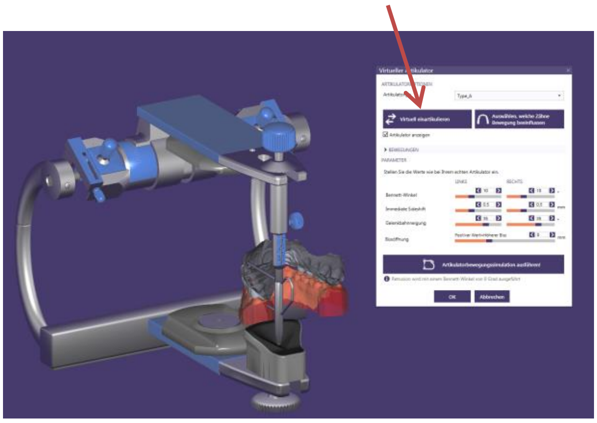

15) Zur besseren Ansicht klicken Sie auf die ☐ neben ***„Artikulator einblenden“*** & ***„Okklusionsebene“***

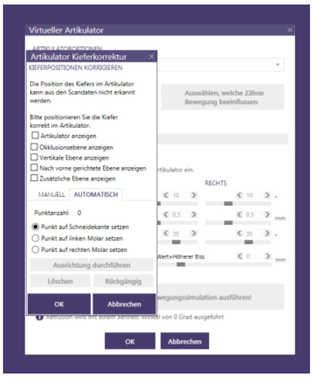

16) Entfernen Sie im Fenster links oben ebenso den **OK + Aufbisschienenteile**

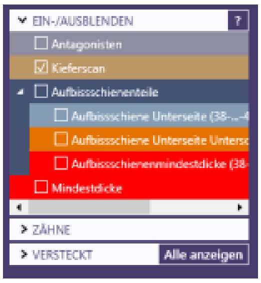

17) Reiter ***„Automatisch“*** auswählen:

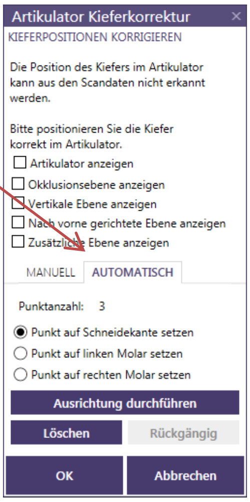

18) Punkte auf **Inzisalpunkt** setzen (Inzisalpunkt zwischen 31+41) sowie auf **Medio-bukkale Höckerspitze** linker Molar (**36**) und rechter Molar (**46**)

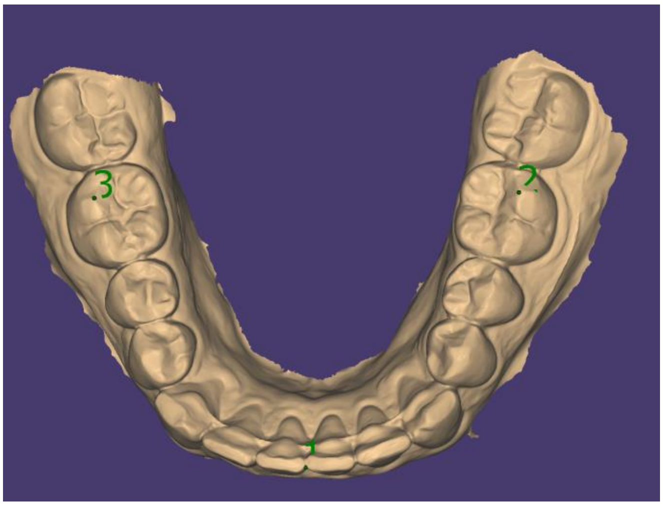

19) Klicken Sie auf ***„Ausrichtung durchführen“***

20) Artikulator + Okklusionsebene Anzeigen -> Ausrichtung kontrollieren:

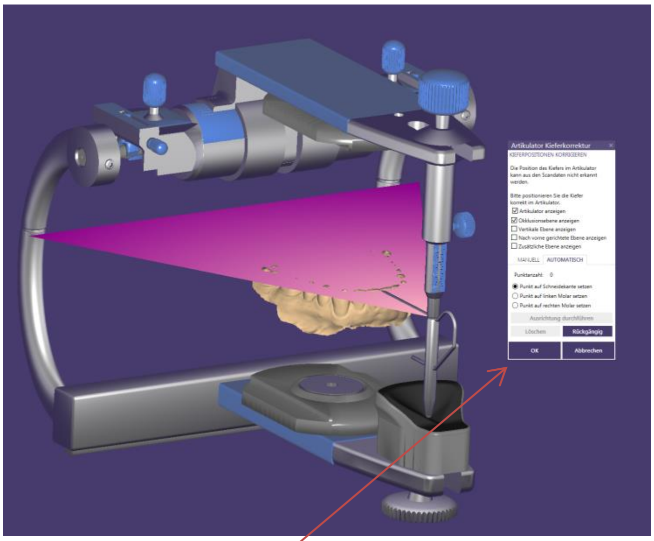

21) Bestätigen mit **OK**

22) Warnmeldung: „Kieferabstand übernehmen“

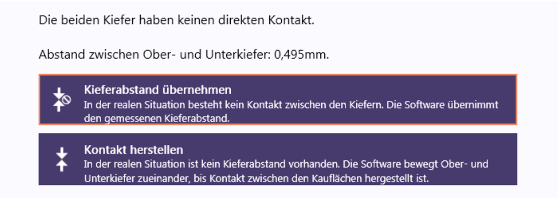

23) Überprüfen Sie die Bisssperrung. Falls der Wachsbiss nicht ausreichend gesperrt hat, muss der Artikulator noch etwas geöffnet werden.

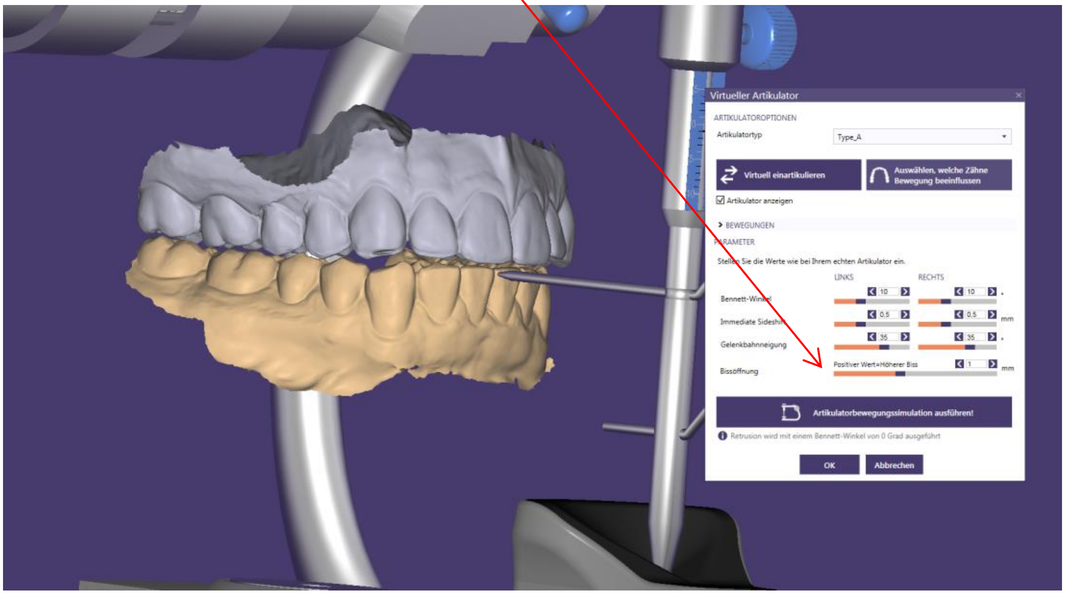

23.1) Über: 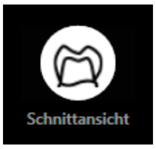

in der rechten Leiste kann der Abstand der Kiefer kontrolliert werden:

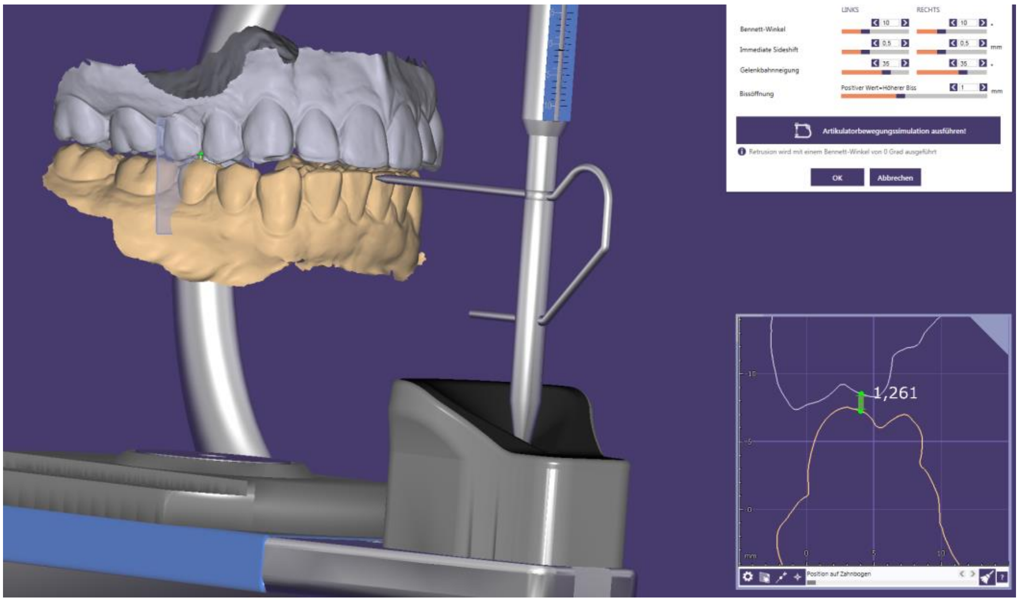

Ziel: ca. 1mm im Molarenbereich -> erneuter Klick auf ***Schnittansicht*** um Fenster zu schließen
(Messen: Auswahl und verschieben von 2 Punkten im Schnittfenster durch Linksklick)
Falls der Artikulator extrem gesperrt werden müsste kann auch ein kleinerer Abstand übernommen werden

24) Optische Kontrolle der Bisssperrung

25) Parameter überprüfen (sollten auf Standarteinstellung belassen werden)

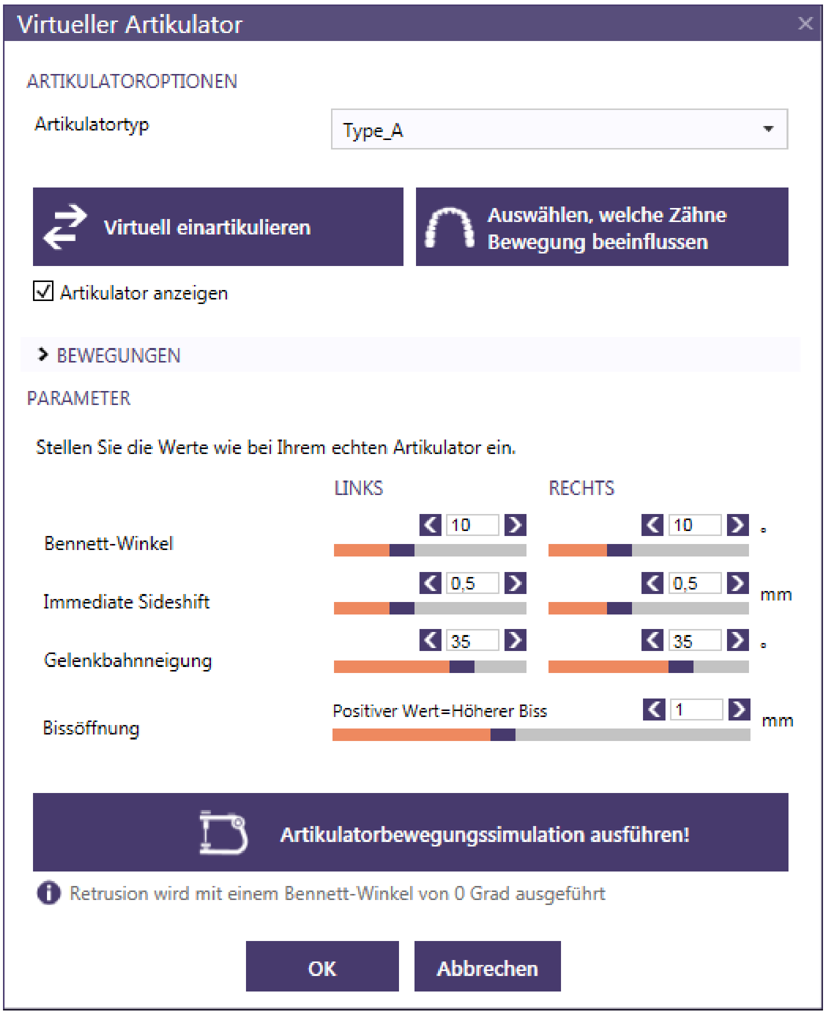

26) und **Artikulatorbewegungssimulation ausführen!**

27) Bestätigen mit **OK**

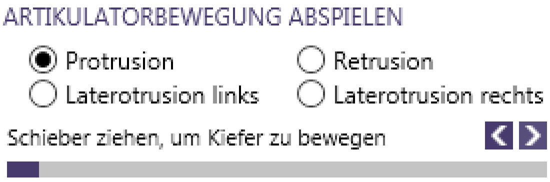

☆ → Neues Fenster oben rechts:

28) Klicken Sie auf 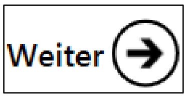

Schienenform:

1) Stellen Sie die Parameter entsprechend ein:

Okklusale Dicke: 2,5mm

Periphere Dicke: 2mm

Glättung: 3mm

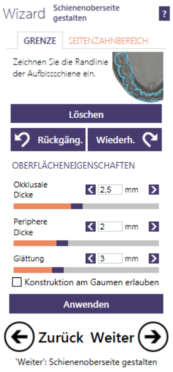

2) Bestimmen Sie die Schienenform durch Festlegen der Schienengrenze

Vestibuläre Gestaltung SZ-Bereich

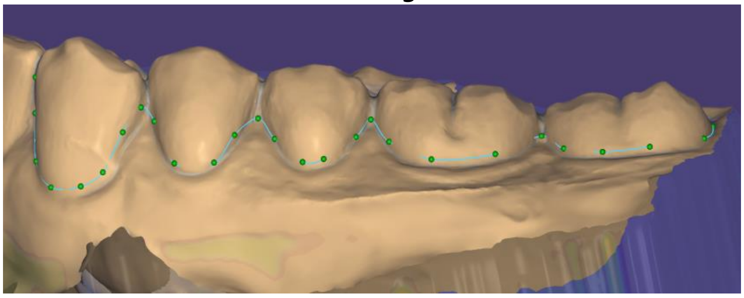

linguale Gestaltung im SZ-Bereich

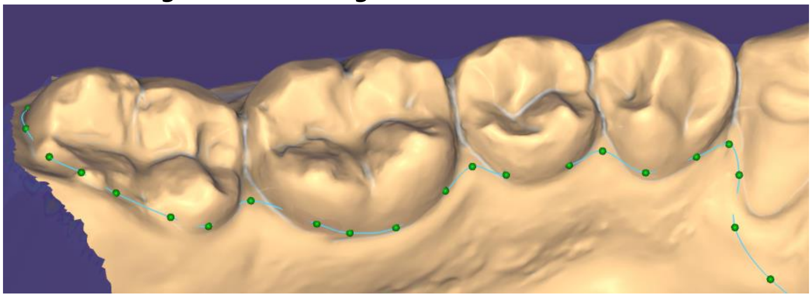

-> nicht bis in den tiefsten Bereich interdental

Frontzahn vestibulär

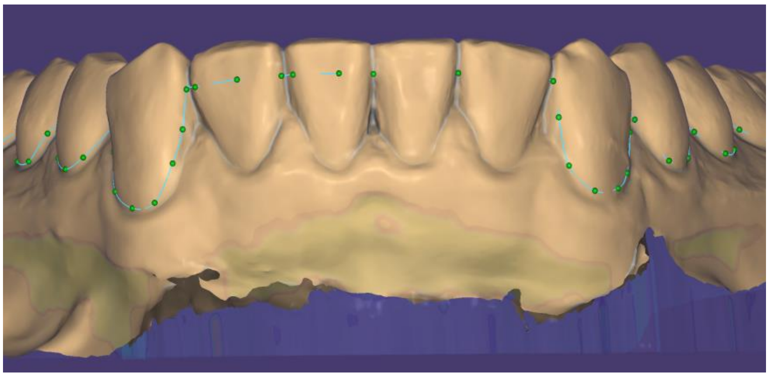

Frontzahn lingual

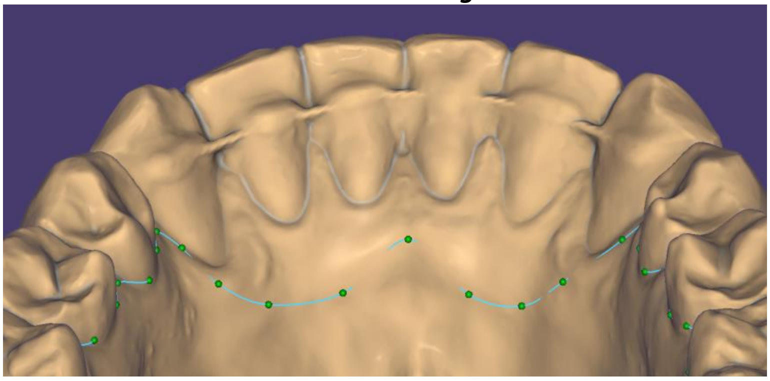

3) Klicken Sie auf „**Anwenden**“

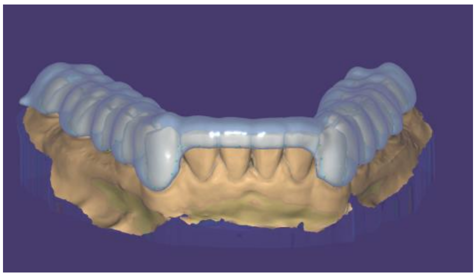

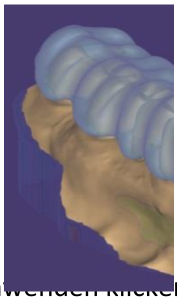

4) Ggf. Verlauf optimieren (danach erneut auf An[illegible]n)

Okklusion anpassen:

(klicken Sie erst auf ***Weiter*** wenn Sie die Anleitung bis zum Schluss durchgearbeitet haben!)

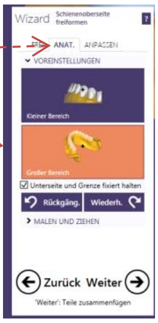

1) Klicken Sie auf den Reiter **„Anat.“** – und wählen **„Großer Bereich“** aus

2) Eckzähne aufbauen:

Vorher: 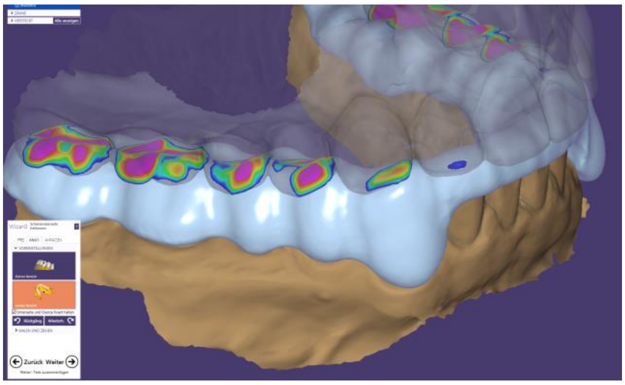

Nacher: 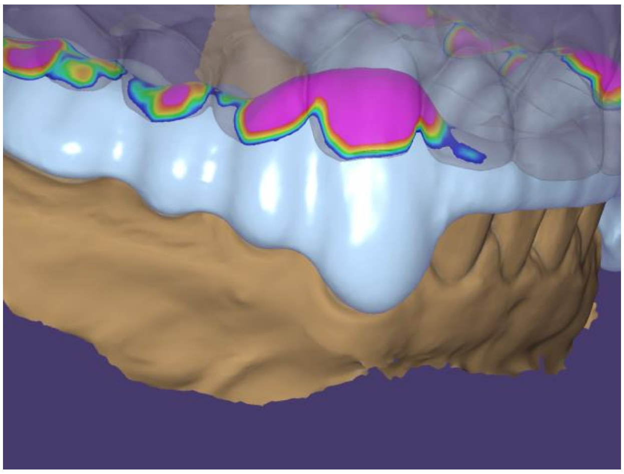

Ziehen Sie mit gedrückter linker Maustaste die Schienenoberfläche, sodass die Eckzähne Kontakt zur Kaufläche haben

3) Bauen sie nun auch lingual das Schild etwas auf (nach lingual ziehen aus okklusaler ansicht)

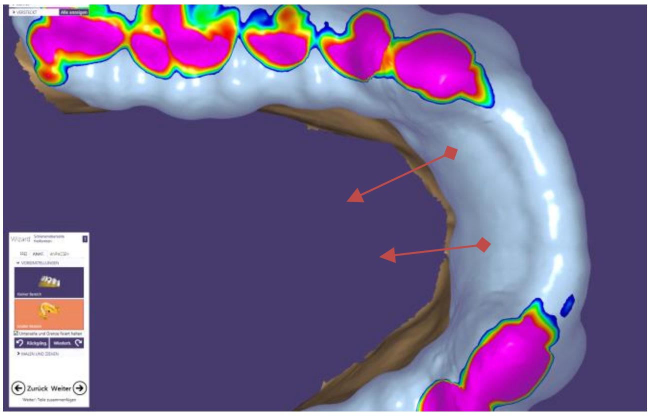

4) Wählen Sie den Reiter -> Anpassen

Nehmen Sie die Einstellung:

Vollanatomie: **0mm**
Okklusionstyp: **dynamisch**

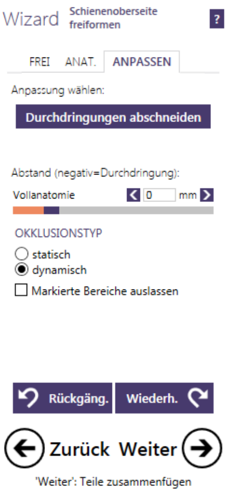

5) Wählen Sie „***Durchdringungen abschneiden***“

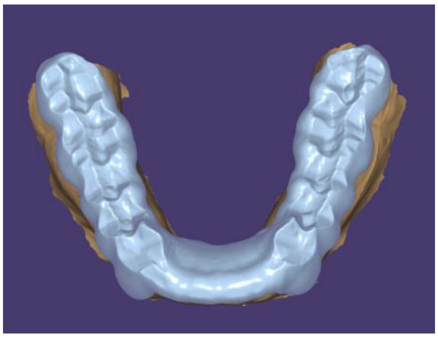

6) Wählen Sie den Reiter ***„Frei“***

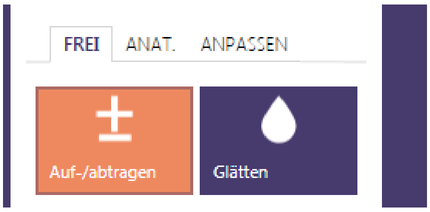

Ziel: -> alle scharfen Kanten glätten und linguale Höcker ebenen

a) Erst Abtragen/Auftragen
b) Glätten (größte Stärke + Pinselgröße)

Danach gezielt von der Seite schauen und aufstehende Schienenanteile gezielt reduzieren und glätten. Ggf. auch aufbauen.

Ziel: Die Kaufläche der Schiene sollte nun möglichst glatt sein und keine steilen Höcker mehr aufweisen

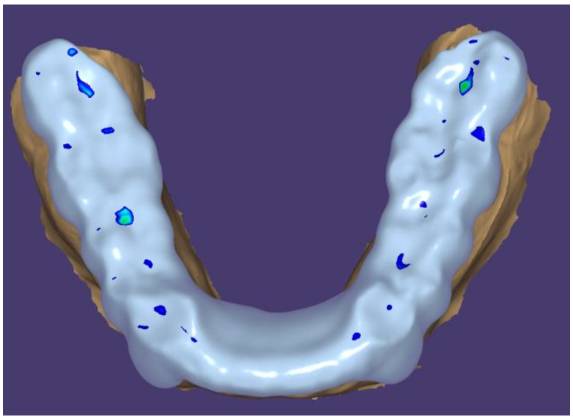

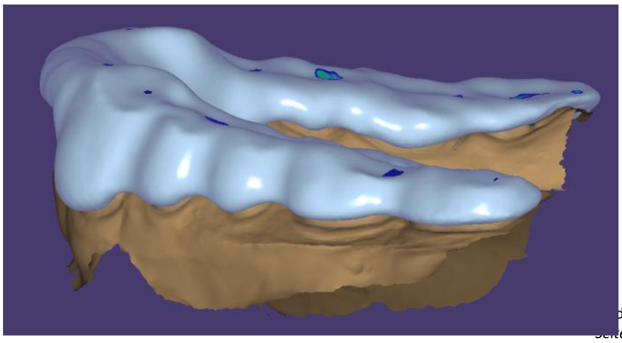

Erneut die Dynamik im Aritkulator ausführen um neue Störflächen zu entfernen:

Nehmen Sie die Einstellung:

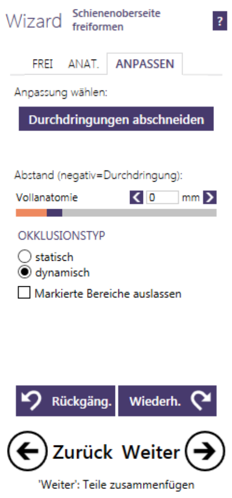

Vollanatomie: **0mm**

Okklusionstyp: **dynamisch**

Wählen Sie „***Durchdringungen abschneiden***“

Statik definieren

1) **OK einblenden auf ca. 80%**
2) **Über Freiformen: „*Auf-/abtragen*“**

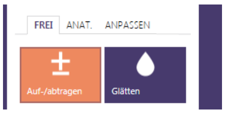

a) Statik auf allen tragenden Höckern im OK in einer Linie aufbauen (Farbumschlag: blau-grün)
b) alle anderen Kontake (so gut es geht) entfernen

Keine Kontakte in der Front 32-42 – 3er sollten leichte Statik haben (für späterer Dynamik)

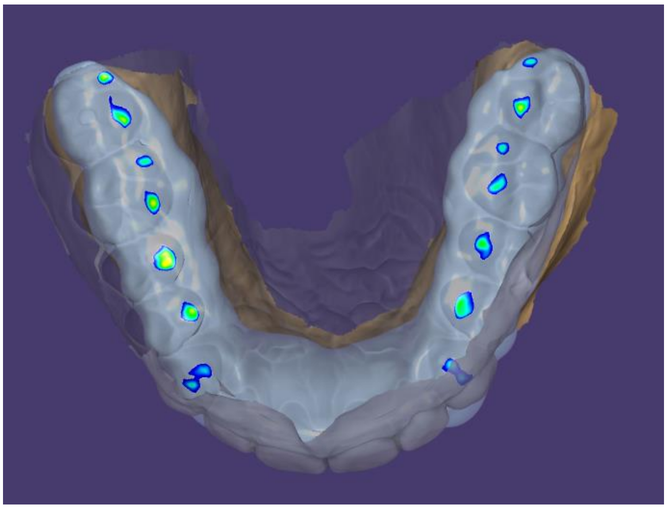

Kontakt-Design: Die Statik sollte im Idealfall so gesetzt werden, dass der Unterkiefer in der Tendenz nach ventral abgleitet:

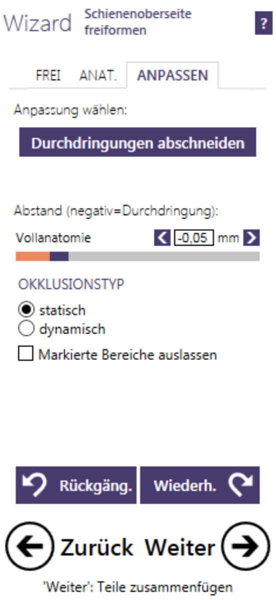

3) Wählen Sie den Reiter ***„Anpassen"***

Vollanatomie: **-0,05mm**

Okklusionstyp: **statisch**

4) ***„Durchdringung abschneiden"*** wählen

➔ Alle Kontakte haben nun die gleiche Kontaktstärke (dunkelblau dargestellt)

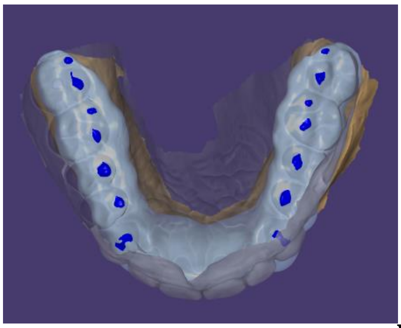

Dynamik:

1) Artikulatorfenster (oben rechts) auf Laterotrusion links stellen:

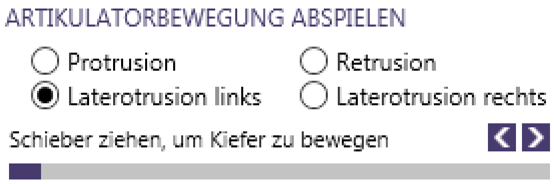

2)

- **a)** 1x auf Pfeil ▶ klicken und Latero-Kontakte anpassen
- **b)** Im Reiter „Anpassen": Durchdringung abschneiden (**statisch** -> **-0,05mm**)
- **c)** Im Reiter „Frei": „Auf-/abtragen" mit dem Pinselwerkzeug
  - i. Fehlende Kontakte aufbauen
  - ii. Störende Kontakte entfernen (*Shift-Taste* gedrückt halten)
- **d)** Im Reiter „Anpassen": Durchdringung abschneiden (**statisch** -> **-0,05mm**)
- **e)** 1x auf Pfeil ▶ klicken
- **f)** …..-> **b)**

☆

Ziel: *Eckzähne,* + in der Initalphase (3-4 x auf den Pfeil) auch die *5er, bis ca. zur Hälfte die 4er,* sollten in der Dynamik mit führen

Alle anderen Kontakte so gut es geht entfernen (Evtl. nicht möglich aufgrund der Unterschreitung der Mindestschichtstärke -> spätere Entfernung an der gedruckten Schiene mit der Fräße nach Anzeichnung im Mund)

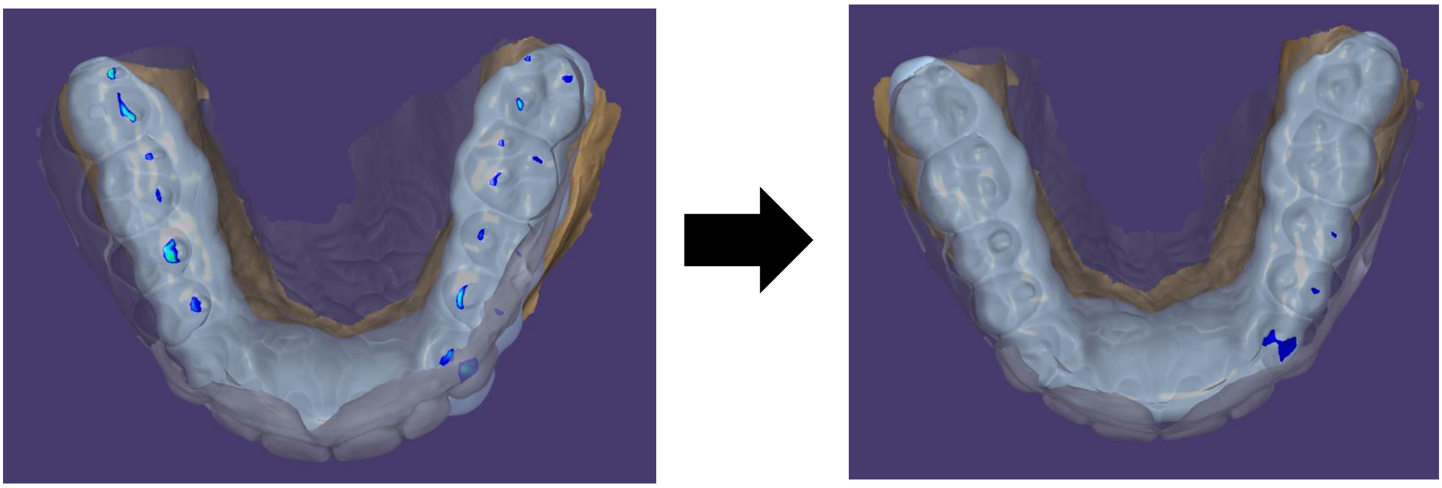

3) Schrittweise bis zum Maximum durchklicken

4) Anschließend einmal Schrittweise zurück klicken und ggf. ab und zu über „Durchdringungen abschneiden“ die Aufbauten kontrollieren und passen.

5) Wenn in der Statik (0 Position) nun vestibulär der 4er Kontakte vorhanden sind -> belassen

6) Gegenseite genauso abarbeiten (Oben rechts: Umschalten auf Laterotrusion rechts)

7) **Abschluss:** Okklusalfläche kontrollieren – Unebenheiten mit bedacht leicht glätten (Glättentool)

8) Lingual Frontzahnbreich glätten

9) Schienenränder ggf. glätten und anpassen.

10) Fertig? Design abschließen mit 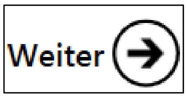

11) **„Ich bin Fertig“**

12) „Weiter“ klicken

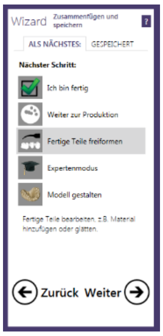

FERTIG ☺

Zeit stoppen und Fragebögen ausfüllen

In Liste eintragen, damit Schiene gedruckt wird.
