# Supplementary material for: Implementation of a Full Digital Workflow by 3D Printing Intraoral Splints Used in Dental Education: An Exploratory Observational Study with Respect to Students’ Experiences
Source: Dent J (Basel). 2022 Dec 26;11(1):5. doi: 10.3390/dj11010005 (PMC9858622; doi:10.3390/dj11010005)
Supplement: Supplementary file 1 [file dentistry-11-00005-s001.zip › Supplement S5- Discarded Questions.pdf]

Overview of discarded questions by questionnaire and reason

**Task**		**Rational to discard questionnaires (N) due to multiple ID notation.**		
**Questionnaire**	**no ID**	**Only one dataset to be kept**	**no dataset to kept**	**Sum discarded**
1 ATP	1			1
1 PTP	4	3	6	13
2 ATP	2			2
3 ATP	1	1	2	4
4 ATP	2	2	2	6
4 ATP	4	8	2	14
**sum**	**14**	**14**	**12**	**40**
