# Supplementary material for: Implementation of a Full Digital Workflow by 3D Printing Intraoral Splints Used in Dental Education: An Exploratory Observational Study with Respect to Students’ Experiences
Source: Dent J (Basel). 2022 Dec 26;11(1):5. doi: 10.3390/dj11010005 (PMC9858622; doi:10.3390/dj11010005)
Supplement: Supplementary file 1 [file dentistry-11-00005-s001.zip › Supplement S6- VAS Questions.pdf]

Table with overview of all VAS responses (percentiles, median, mean, SD, N)

Questionnaire	Question	VAS 0	VAS 100	25th percentile	Median	75th percentile	SD	N
Intraoral scan	How do you evaluate the handling of the intraoral scanner?	*simple*	*difficult*	8	21	34	19,7	77
	How do you rate the amount of time required for the scan?	*swift*	*long-winded*	15	27	52	23,4	74
	Were you adequately prepared for independent scanning after the lecture with a demo?	*fully agree*	*totally disagree*	7	15	33	18,7	77
	How would you rate the demonstration and lecture on IO scanning?	*helpful*	*unnecessary*	2	8	19,75	12,3	76
	How would you rate the integration of intraoral scanning in undergraduate dental education?	*helpful*	*unnecessary*	1,75	7	18	16,4	78
	How did you get along with the scan time/number of images?	*very well*	*unsatisfactory*	7	20	33	21,4	78
	How did you feel during the scanning process?	*pleasant*	*unpleasant*	17,75	30,5	48,25	19,5	66
	Did you feel a gagging sensation during the scanning process?	*none*	*pronounced*	0	3	15	21,3	66
	Did you have the feeling that you had to open your mouth very wide during the scan?	*fully agree*	*totally disagree*	18,75	47	69	29,8	66
	How do you rate the amount of time required for the scan?	*swift*	*long-winded*	12	24,5	50	27,3	66
Computer-aided design	Were you adequately prepared for the splint design after preparation (demonstration / instruction)?	*fully agree*	*totally disagree*	9	22,5	39,5	21,9	70
	How do you rate the time required for the splint design (CAD)?	*swift*	*long-winded*	38	62	75	24,5	67
	I can perform the splint design independently	*fully agree*	*totally disagree*	18,5	37	60,5	24,6	69
	Which method would you prefer for the training?	*digital*	*conventional*	19,5	43	74,5	32,3	69
Finishing	Properties of the splint material to be processed	*simple*	*difficult*	12,25	23	30	18,9	56
	How do you evaluate the time and effort for the elaboration?	*simple*	*difficult*	12,75	27	37,25	22,4	62
	How do you rate your final result?	*very good*	*very poor*	14	27	34	22,3	62
	How do you rate the polishing?	*very good*	*very poor*	18,5	26,5	40,25	20,4	62
	How do you rate the effort for polishing?	*very little*	*very high*	20	29	49,5	23,1	62
Insetion	The initial fit of the splint is:	*very good*	*very poor*	24	44	72	28,7	57
	How do you rate the effort to obtain an equilibrated bite plane?	*very low*	*very high*	27,25	44	68,75	24,7	56
	How do you rate the final result?	*very good*	*very poor*	17	26	46	20,1	55
	Do you notice a feeling of tension when inserting/wearing the splint	*initial*	*permanent*	0	0	60,25	37,6	50
	How is the feeling with the splint fitted in the mouth (without occlusion)	*comfortable*	*uncomfortable*	18	31,5	56	25	50
	I have noticed roughness on the splint	*fully agree*	*totally disagree*	5,25	11	24,5	21,5	52
	Wearing the splint is (wearing comfort + occlusion):	*comfortable*	*uncomfortable*	13	33,5	56,75	27,6	48
