# Supplementary material for: Implementation of a Full Digital Workflow by 3D Printing Intraoral Splints Used in Dental Education: An Exploratory Observational Study with Respect to Students’ Experiences
Source: Dent J (Basel). 2022 Dec 26;11(1):5. doi: 10.3390/dj11010005 (PMC9858622; doi:10.3390/dj11010005)
Supplement: Supplementary file 1 [file dentistry-11-00005-s001.zip › Supplement S7- MC SC Questions.pdf]

**Table with overview of all Multiple Choice and Single Choice questions (N, %)**

## Intraoral Scan

| How many attempts did you need? (SC) | % | N |
|---|---|---|
| 1 | 69,23% | 54 |
| 2 | 24,36% | 19 |
| 3 | 5,13% | 4 |
| 5 | 1,28% | 1 |

| When comparing the conventional impression with the intraoral scan - Which procedure do you prefer? (SC) | % | N |
|---|---|---|
| both equal | 31,6% | 24 |
| intraoral scan | 47,4% | 36 |
| conventional impression | 21,1% | 16 |

| Were there any difficulties in using the scanner? (MC) | % | N |
|---|---|---|
| none | 57,7% | 45 |
| yes: | 42,3% | 33 |
| *Software operation* | *6,4%* | *5* |
| *Scanner operation* | *15,4%* | *12* |
| *Scan sequence (Upper jaw/lower jaw/ bite)* | *5,1%* | *4* |
| *scan over 1500 pictures* | *23,1%* | *18* |

| How often did you pause the scan for a single jaw scan? (SC) | % | N |
|---|---|---|
| 0-3 | 39,7% | 31 |
| 3-5 | 55,1% | 43 |
| 5-10 | 3,9% | 3 |
| >10x | 1,3% | 1 |

| How did you feel after the scan? (MC) | % | N |
|---|---|---|
| without change | 60,6% | 40 |
| changed: | 39,4% | 26 |
| *muscle strain* | *18,2%* | *12* |
| *mouth pain* | *6,1%* | *4* |
| *dry mouth* | *25,8%* | *17* |
| *changed okklusion* | | |
| *change in tase* | *9,1%* | *6* |
| *foreign body sensation* | *1,5%* | *1* |

## Computer aided Design

| The construction of the splint was: (SC) | % | N |
|---|---|---|
| self-explaining | 22,9% | 16 |
| challenging | 61,4% | 43 |
| difficult | 12,9% | 9 |
| exceedingly difficult | 2,9% | 2 |

| Were there areas in the scan that were not adequately captured? (SC) | % | N |
|---|---|---|
| no | 85,51% | 59 |
| yes | 14,49% | 10 |

| Was assistance necessary? (SC) | % | N |
|---|---|---|
| no | 33,3% | 23 |
| slightly | 49,3% | 34 |
| a lot | 13,0% | 9 |
| only possible with assistance | 4,4% | 3 |

| Did you have to restart the splint design several times? (SC) | % | N |
|---|---|---|
| no | 71,43% | 50 |
| 2 | 25,71% | 18 |
| 3 | 2,86% | 2 |

| Have any problems occurred during splint design (SC) | % | N |
|---|---|---|
| no | 53,73% | 36 |
| yes | 46,27% | 31 |

| Have you worked with CAD designers before? (SC) | % | N |
|---|---|---|
| not surveyed in WS1718 | 30,43% | 21 |
| no | 11,59% | 8 |
| yes | 57,97% | 40 |

## Finishing

| Properties of the splint material to be processed (MC) | % | N |
|---|---|---|
| familiar | 66,7% | 42 |
| unfamiliar | 17,5% | 11 |
| milling clog | 6,4% | 4 |
| soft - much remove | 31,8% | 20 |
| hard - less remove | 12,7% | 8 |
| polishes well | 46,0% | 29 |
| does not polish well | 25,4% | 16 |
| streaking | 4,8% | 3 |
| edges breaking off | 6,4% | 4 |
| low working pressure required | 49,2% | 31 |
| high working pressure required | 7,9% | 5 |

| Have problems occurred during finishing? (SC) | % | N |
|---|---|---|
| no | 79,66% | 47 |
| yes | 20,34% | 12 |

## Table with overview of all Multiple Choice and Single Choice questions (N, %)

# Insertion

| The initial fit of the splint is: (MC) | % | N |
|---|---|---|
| ***wobbling*** | **16,7%** | **10** |
| ***tension*** | **53,3%** | **32** |
| **pressure on the gingiva** | **0,0%** | **0** |
| **jamming** | **26,7%** | **16** |

| The initial fit of the splint is: (SC) | % | N |
|---|---|---|
| clinical acceptable | **68,52%** | **37** |
| clinical unacceptable | **31,48%** | **17** |

| The initial fit of the splint is correctable (SC) | % | N |
|---|---|---|
| not required | **65,45%** | **36** |
| yes | **27,27%** | **15** |
| no | **7,27%** | **4** |

| The retention of the splint (against detachment from end position) (SC) | % | N |
|---|---|---|
| clinical acceptable | **62,71%** | **37** |
| too loose | **13,56%** | **8** |
| too tight | **23,73%** | **14** |

| The retention of the splint (against detachment from end position) is correctable (SC) | % | N |
|---|---|---|
| not required | **66,07%** | **37** |
| yes | **23,21%** | **13** |
| no | **10,71%** | **6** |

| Obtaining an equilibrated bite plane? (SC) | % | N |
|---|---|---|
| possible | **91,80%** | **56** |
| not possible | **8,20%** | **5** |

| Final result: Splint insertable? (SC) | % | N |
|---|---|---|
| yes | **88,52%** | **54** |
| no | **11,48%** | **7** |

| Do you notice a feeling of tension when inserting/wearing the splint (SC) | % | N |
|---|---|---|
| no | **42,00%** | **21** |
| yes | **24,00%** | **12** |
| not surveyed WS1718 | **34,00%** | **17** |

| How do you describe the taste when you first insert the splint: (MC) | % | N |
|---|---|---|
| **neutral** | **55,8%** | **29** |
| **toxic** | **9,6%** | **5** |
| **chemical** | **30,8%** | **16** |
| **sweet** | **1,9%** | **1** |
| **sour** | **1,9%** | **1** |
| **foul** | **0,0%** | **0** |
| **other:** | **11,5%** | **6** |
| *long after disinfection* | ***1,9%*** | ***1*** |
| *plastic* | ***5,7%*** | ***3*** |
| *undefinable* | ***1,9%*** | ***1*** |
| *Polishing agents* | ***1,9%*** | ***1*** |

| Correction of splint for wearing (wearing comfort + occlusion): (SC) | % | N |
|---|---|---|
| no correction neccesary | **72,00%** | **36** |
| correctable | **8,00%** | **4** |
| uncorrectable | **20,00%** | **10** |

| How do you rate the overall workflow with scanning, designing, finishing, and inserting the splin (SC) | % | N |
|---|---|---|
| **very good** | **6,9%** | **4** |
| **good** | **36,2%** | **21** |
| **satisfactory** | **36,2%** | **21** |
| **adequate** | **13,8%** | **8** |
| **inadequate** | **6,9%** | **4** |
