# Supplementary material for: Implementation of a Full Digital Workflow by 3D Printing Intraoral Splints Used in Dental Education: An Exploratory Observational Study with Respect to Students’ Experiences
Source: Dent J (Basel). 2022 Dec 26;11(1):5. doi: 10.3390/dj11010005 (PMC9858622; doi:10.3390/dj11010005)
Supplement: Supplementary file 1 [file dentistry-11-00005-s001.zip › Supplement S8- Comparing the mean values of responses to time-relevant items in each cohort.pdf]

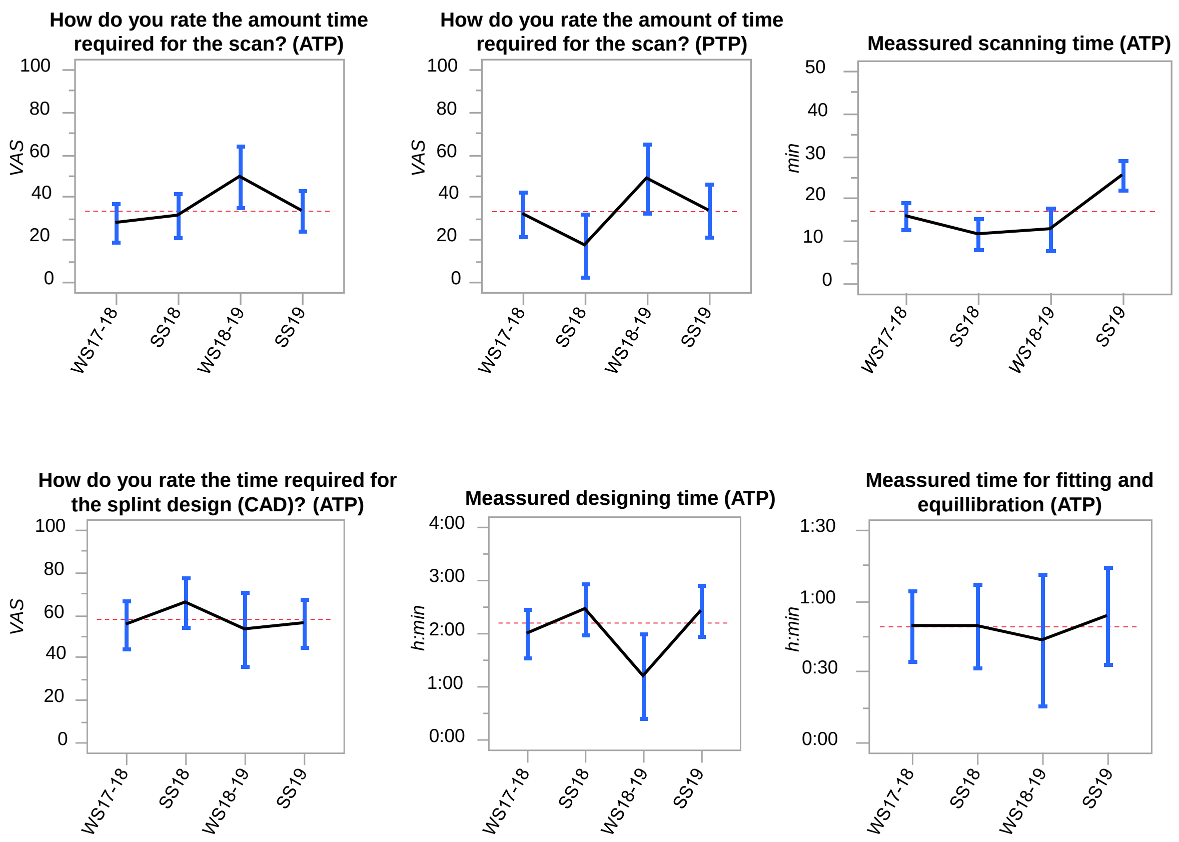

Comparing the mean values of responses to time-relevant items in each cohort. The cohorts (x-axis) and values of items scale are descripted by the least squares mean values (y-axis) with standard deviations (blue bars) and the mean of means (horizontal dotted line).
