# Supplementary material for: Implementation of a Full Digital Workflow by 3D Printing Intraoral Splints Used in Dental Education: An Exploratory Observational Study with Respect to Students’ Experiences
Source: Dent J (Basel). 2022 Dec 26;11(1):5. doi: 10.3390/dj11010005 (PMC9858622; doi:10.3390/dj11010005)
Supplement: Supplementary file 1 [file dentistry-11-00005-s001.zip › Supplement S9- Comparison of mean values of VAS and duration by impression method preference.pdf]

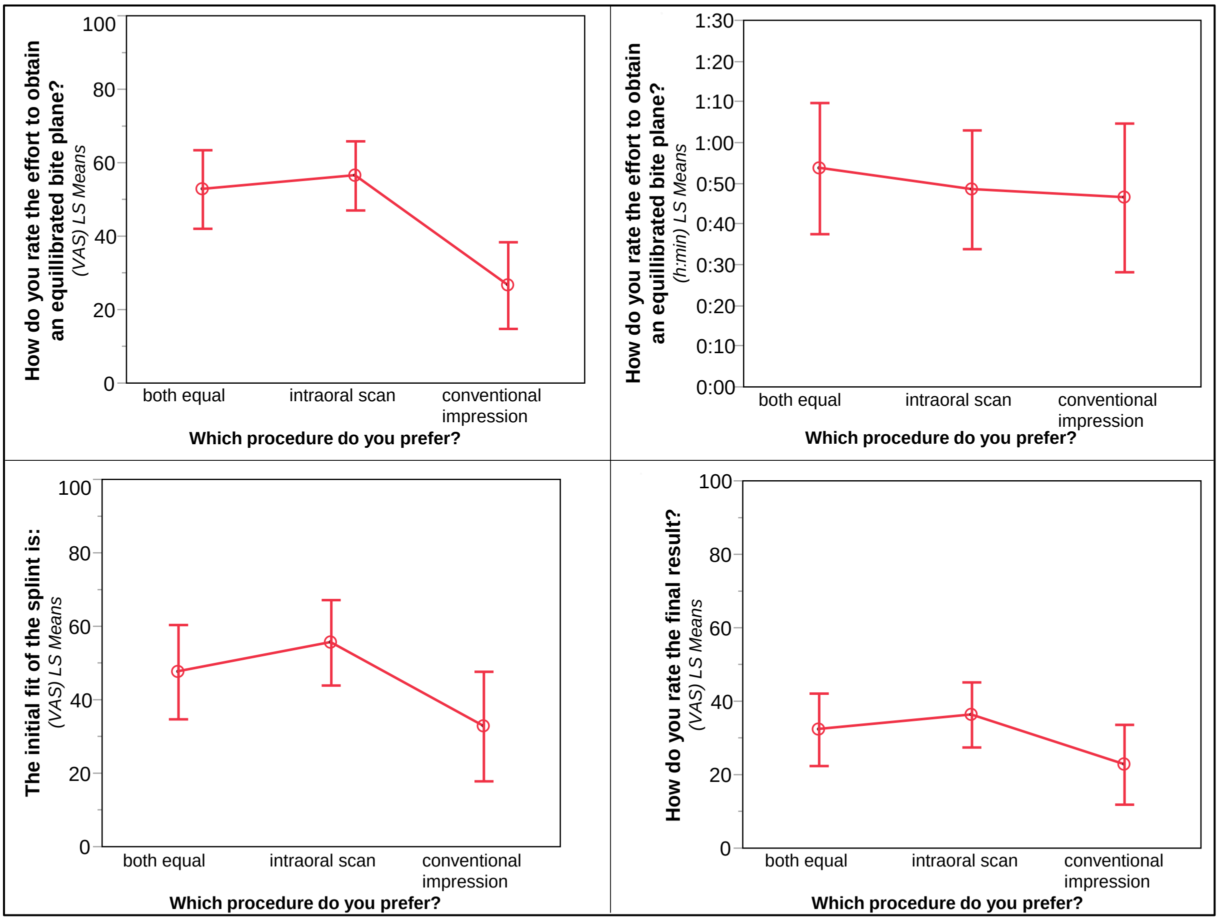

Figure: Comparison of mean values of VAS and duration by impression method preference for effort (sensation and time) to produce an equilibrated bite splint and evaluate initial fit and finished workpiece.
